# Supplementary material for: Extracellular matrix remodeling through endocytosis and resurfacing of Tenascin-R
Source: Nat Commun. 2021 Dec 8;12:7129. doi: 10.1038/s41467-021-27462-7 (PMC8654841; doi:10.1038/s41467-021-27462-7)
Supplement: Supplementary file 1 — Supplementary Information [file 41467_2021_27462_MOESM1_ESM.pdf]

SUPPLEMENTARY INFORMATION

Extracellular matrix remodeling through endocytosis and resurfacing of Tenascin-R

Tal M. Dankovich[1,2*], Rahul Kaushik[3,4], Linda H.M. Olsthoorn[2,5], Gabriel Cassinelli Petersen[1], Philipp Emanuel Giro[1], Verena Kluever[1], Paola Agüi-Gonzalez[1], Katharina Grewe[1], Guobin Bao[1,6], Sabine Beuermann[7], Hannah Abdul Hadi[1], Jose Doeren[1], Simon Klöppner[1], Benjamin H. Cooper[7], Alexander Dityatev[3,4,8], Silvio O. Rizzoli[1,9*]

Affiliations
[1]University Medical Center Göttingen, Institute for Neuro- and Sensory Physiology, Excellence Cluster Multiscale Bioimaging, Göttingen, Germany.
[2]International Max Planck Research School for Neuroscience, Göttingen, Germany.
[3]Molecular Neuroplasticity, German Center for Neurodegenerative Diseases (DZNE), Magdeburg, Germany.
[4]Center for Behavioral Brain Sciences (CBBS), Magdeburg, Germany.
[5]Max Planck Institute for Biophysical Chemistry, Göttingen, Germany.
[6]University Medical Center Göttingen, Institute of Pharmacology and Toxicology, Göttingen, Germany.
[7]Max Planck Institute for Experimental Medicine, Göttingen, Germany.
[8]Medical Faculty, Otto von Guericke University, Magdeburg, Germany.
[9]Biostructural Imaging of Neurodegeneration (BIN) Center, Göttingen, Germany.
***Correspondence**: Tal M. Dankovich: tal.dankovich@med.uni-goettingen.de, Silvio O. Rizzoli: srizzol@gwdg.de

Included in this PDF:
Supplementary Figure 1-20
References for Supplementary Figures 1-20

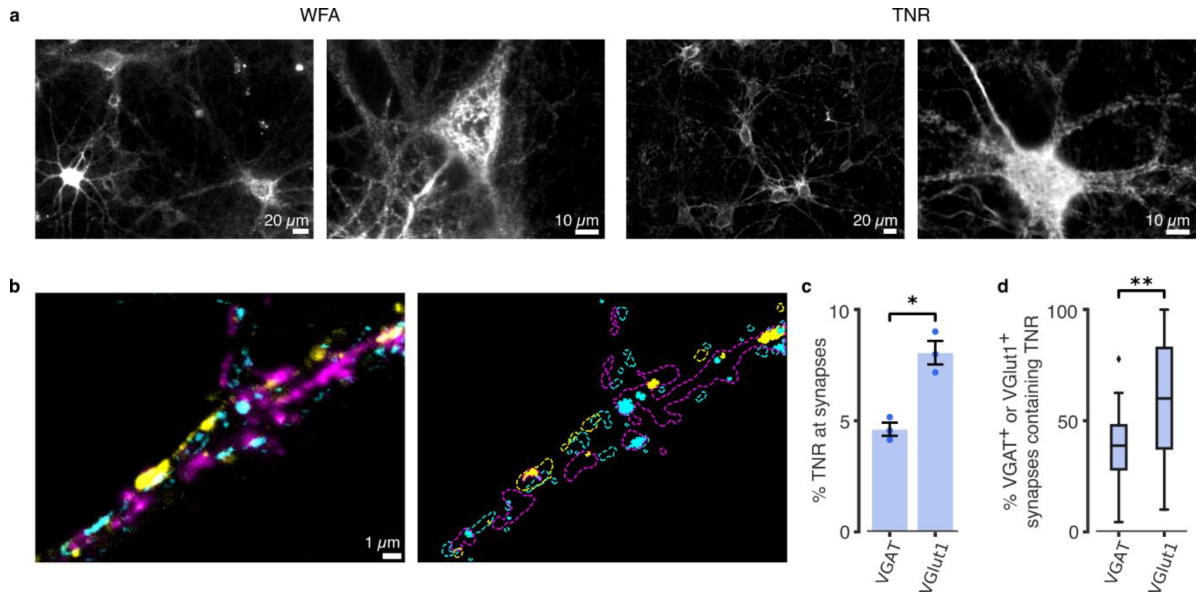

Supplementary Figure 1 Antibody-labeled TNR molecules are present in the perineuronal net and at synapses, and are extremely stable. a, We fixed neuronal cultures at 14 days *in vitro* (DIV14), and we then labeled them with *Wisteria floribunda* agglutin (WFA), which binds chondroitin-sulfate (CS)-bearing proteoglycans [1] (left), or with TNR antibodies (right), and imaged them with epifluorescence microscopy. Both labels exhibited lattice-like structures that surrounded the somas and proximal dendrites of a subset of neurons (perineuronal nets; PNNs), which also suggests that the ECM has reached a sufficient level of maturity in these cultures. Scale bar = 20 µm (left panels), 10 µm (right panels). N = 3 independent experiments. **b**, To assess the amount of TNR at excitatory and inhibitory presynaptic boutons, we labeled all TNR epitopes by incubating live neurons with fluorophore-conjugated TNR antibodies (magenta). Glutamate-releasing (excitatory) and GABA-releasing (inhibitory) boutons were determined by immunostaining the synaptic vesicle markers VGlut1 (yellow) and VGAT (cyan), respectively. The right panel shows the boundaries of the boutons and of TNR domains. The regions where high intensities of TNR and synaptic signals overlap are indicated by full shading in yellow or cyan. TNR, VGlut1 and VGAT are imaged in confocal microscopy. Scale bar: 1 µm. **c-d**, We determined the colocalization of TNR with VGlut1- and VGAT-positive presynapses. We measured the amount of TNR in fully colocalizing pixels, as a percentage of the total TNR staining in the images (panel c). A higher proportion of all TNR epitopes can be found at excitatory synapses. Data represent the mean ± SEM, with dots indicating independent experiments. To determine the proportion of synapses that overlap with TNR signal, we thresholded the VGlut1/VGAT channels to identify individual synapses, and determined the percentage of these synapses that overlap with TNR signal (panel d). We found that the percentage of TNR+/VGlut1+ synapses (~60%) is significantly higher than the percentage of TNR+/VGAT+ synapses (~40%). Boxes show median (mid-line) and quartiles, and whiskers show minimum and maximum values. Outliers were omitted according to inter-quartile range (IQR) proximity (exceeding 1.5*IQR). N = 3 experiments, 15 neurons imaged per experiment.Statistical significance was evaluated using two-sided paired *t*-tests: t = 4.684, *p = 0.043 (panel c); t = 3.312, **p = 0.003 (panel d).

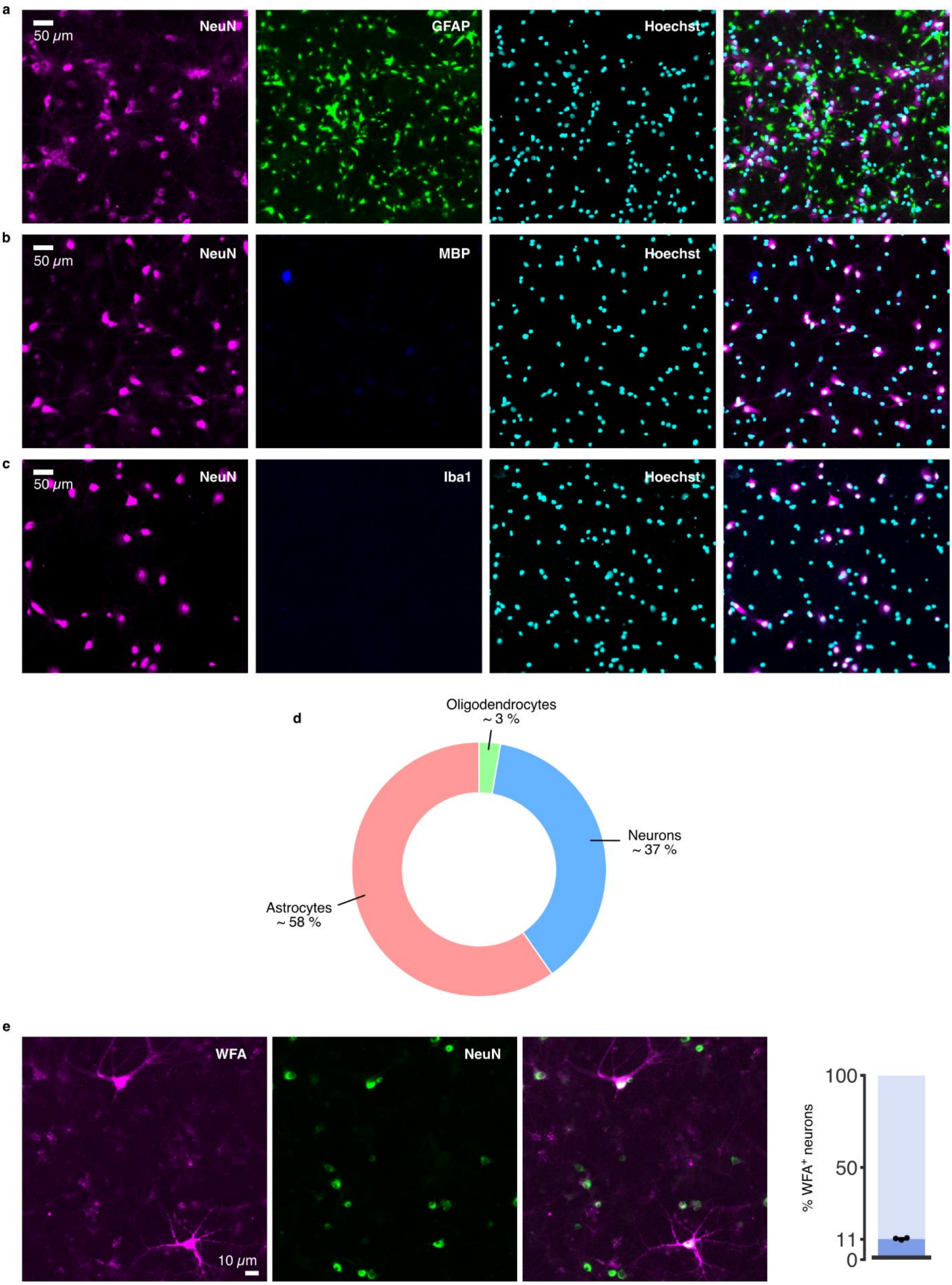

Supplementary Figure 2 Characterization of the dissociated neuronal cultures used in the study. a-d, To determine the fraction of different cell types in our cultures at 14 DIV, we performed a co-immunostaining with different cell-specific markers, and imaged large fields of view in epifluorescence microscopy. We counted cells overlapping with NeuN, GFAP (panel a), myelin basic protein (MBP) (panel b) or Iba1 (panel c) signals to quantify the number of neurons, astrocytes, oligodendrocytes and microglia, respectively. The cell nuclei were identified using a Hoechst staining. Scale bar = 50 μm. **d**, A quantification of the mean fraction of each cell type

in the culture. N = 3 independent experiments. **e**, To determine the proportion of PNN-associated neurons at 14 DIV, we fixed neurons and stained them with WFA (magenta), to detect PNNs, and with NeuN antibodies (green), to label all neurons. We then imaged large fields of view with epifluorescence microscopy and counted the number of WFA+ neurons. We determined the proportion of PNN-associated neurons in our cultures to be ~11%. N = 3 individual experiments, 10 images per datapoint. Data represent the mean ± SEM, with dots indicating independent experiments.

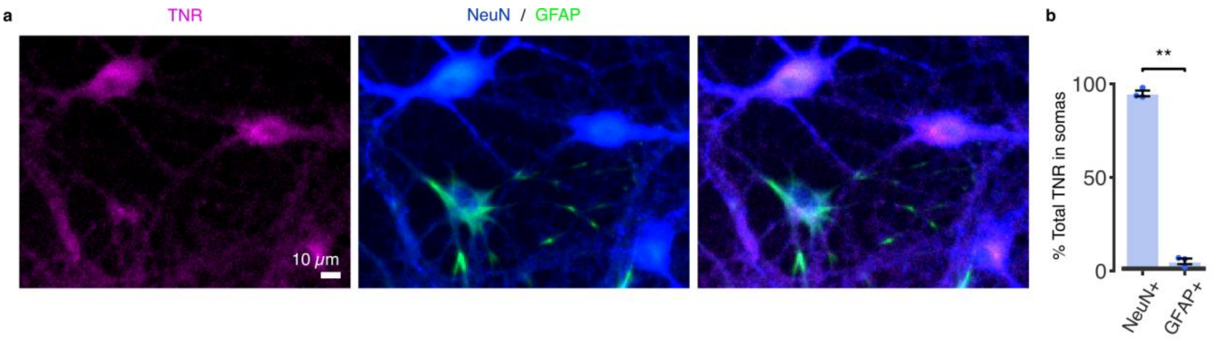

Supplementary Figure 3 A minimal amount of internalized TNR is found in astrocytes. We checked whether recycling TNR epitopes could be detected within astrocytes by co-immunostaining cultures with the astrocytic marker GFAP and the neuronal marker NeuN (using an antibody that recognizes multiple isoforms of the RNA-binding protein FOX-3, localizing to both the nuclei and the cytoplasm [2]). This staining reveals very strongly neuronal cell bodies and the main neurites of neurons, with only very low background in astrocytes. **a**, We labeled newly-emerged TNR epitopes 12 hours post-blocking, by applying fluorophore-conjugated antibodies for 1 hour. After a further incubation of 12 hours, to allow for internalization, we stripped the surface-bound epitopes using proteinase K. We then fixed the neurons and immunostained them with GFAP and NeuN to distinguish astrocytes and neurons, and imaged these with epifluorescence microscopy. The TNR signal was predominantly present in NeuN+/GFAP- cells, and was significantly weaker in GFAP+ cells. Scale bar = 10 µm. **b**, We quantified the TNR signal in NeuN+/GFAP- and GFAP+ regions of interest as a percentage of the total TNR staining in the images. We found that only ~5% of the internalized TNR could be detected in GFAP+ cells, confirming that this process occurs predominantly in neurons. N = 3 independent experiments, at least 10 images per datapoint. Statistical significance was evaluated using a two-sided paired *t*-test ($t = 29.49$, $**p = 0.001$). Data represent the mean ± SEM, with dots indicating individual experiments.

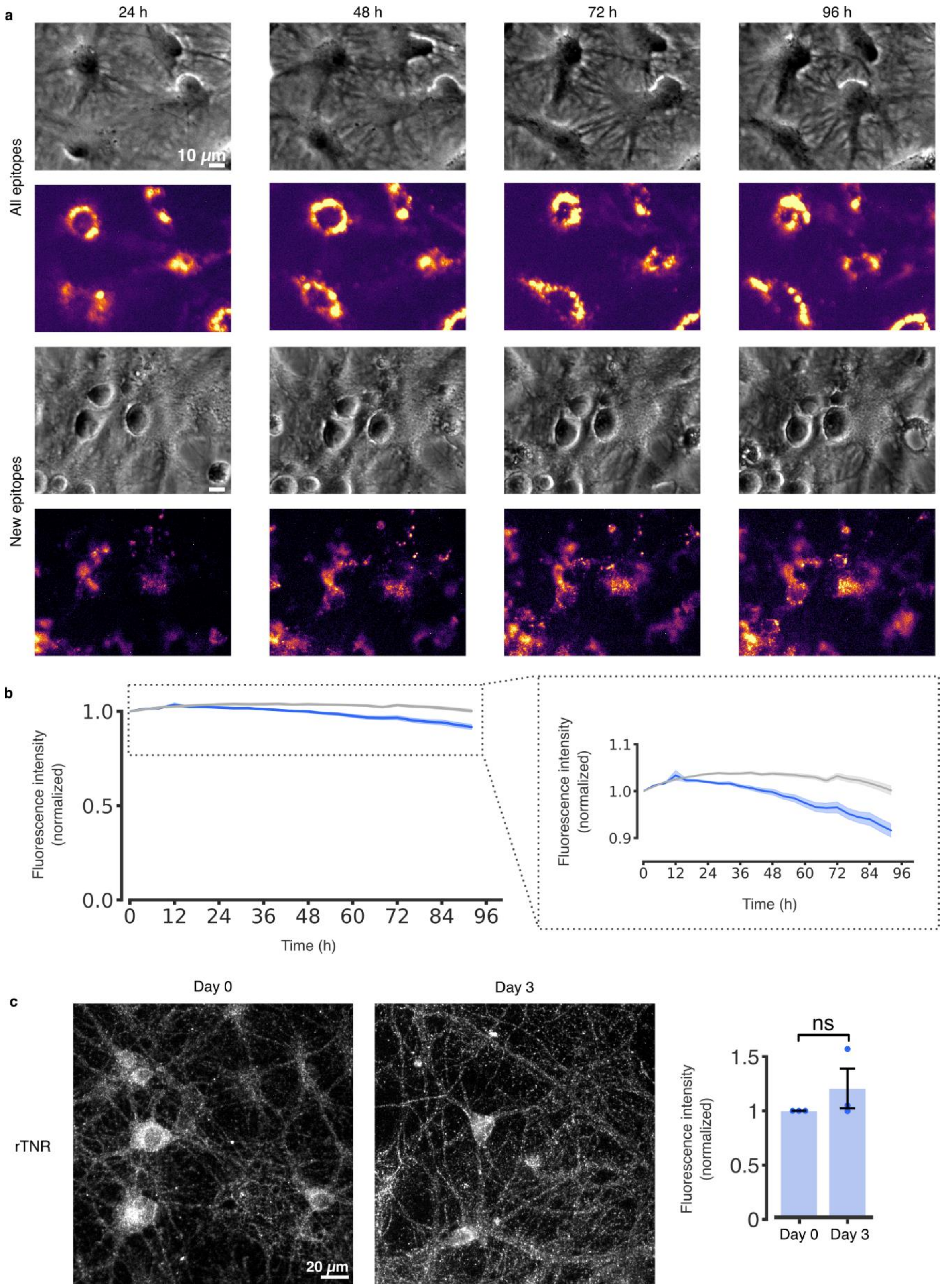
a
24 h
48 h
72 h
96 h
All epitopes
New epitopes
10 μm
b
Fluorescence intensity (normalized)
Time (h)
c
Day 0
Day 3
rTNR
20 μm
ns
Fluorescence intensity (normalized)
Day 0
Day 3

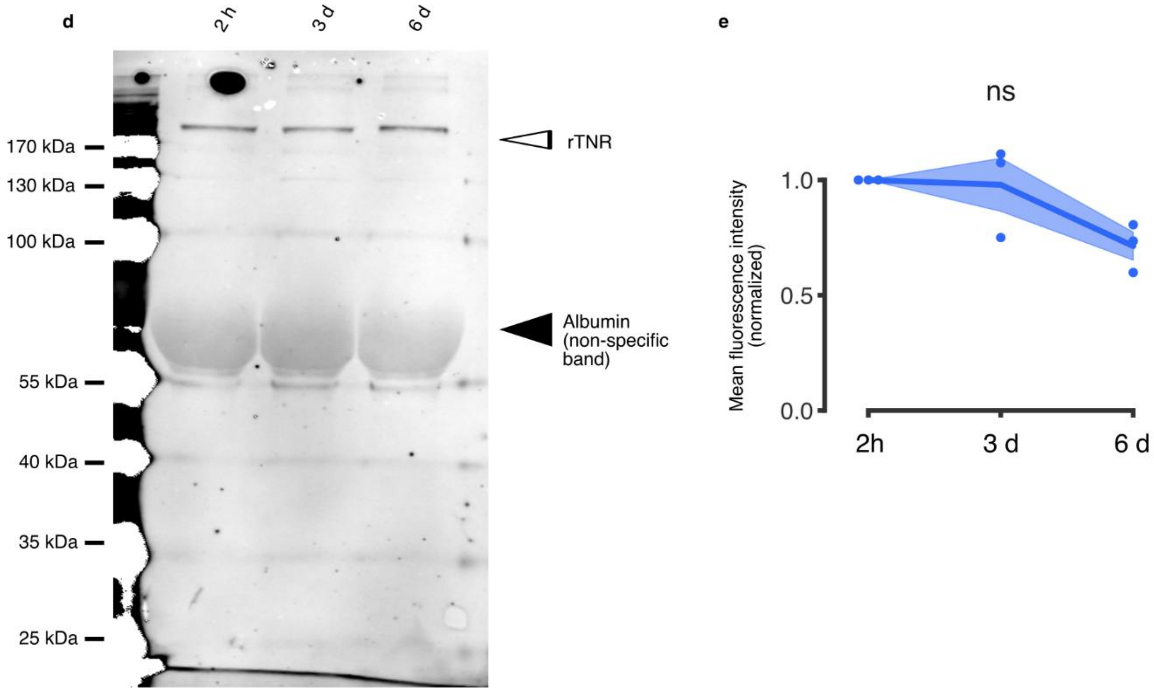

Supplementary Figure 4 TNR is degraded slowly in neuronal cultures. a-b, Either all TNR molecules or newly-emerged TNR molecules (4 hours post-blocking) were labeled by applying fluorophore-conjugated TNR antibodies for 1 hour. We then imaged the neurons for up to 96 hours using an automated cell incubator/microscope setup (BioSpa Live Cell Analysis System, BioTek, USA). **a**, Top panels: phase-contrast images of a single field of view after 1, 2, 3 and 4 days. Bottom panels: the corresponding images in the fluorescent channel. Scale bar = 10 µm. **b**, The graph shows the mean fluorescence intensity, analyzed over the entire images, normalized to the t_0 timepoint. A very small overall loss of signal is seen, confirming the slow degradation and turnover of TNR, as known from the literature. Blue and gray lines represent new and all epitopes, respectively. N = 3 independent experiments, >100 neurons imaged per experiment. Data represent the mean (line) ± SEM (shaded region). **c**, We pulsed the neuronal cultures with a His-tagged recombinant TNR (rTNR), and fixed the neurons immediately after or following a 3-day incubation. We then immunostained the neurons with antibodies against His tag and imaged these with epifluorescence microscopy. Neurons show similar amounts of rTNR at day 0 and day 3 following the pulse. Scale bar = 20 µm. A quantification of the mean fluorescence intensity expressed as fold over background, normalized to the ‘day 0’ condition, confirmed that there is no significant degradation of rTNR after 3 days. N = 3 independent experiments, 5 images per datapoint. Statistical significance was evaluated using a two-sided paired *t*-test (t = 1.126, $p = 0.377$). Data represent the mean ± SEM, with dots indicating independent experiments. **d**, We assessed the amounts of rTNR after 2 hours, 3 days and 6 days of incubation with neuronal cultures using Western blotting. A typical example is shown here. **e**, A quantification of the rTNR band intensities (normalized), demonstrates only a minor reduction of rTNR. This is in line with the long lifetime of TNR in cultures (half-life of ~7 days [3]). N = 3 independent experiments. Statistical significance was assessed using repeated-measures one-way ANOVA ($F_{1.045, 2.09} = 7.565$, $p = 0.106$). Data represent the mean (lines) ± SEM (shaded regions); dots indicate the individual experiments. The black arrowhead indicates the large non-specific band induced by the presence of albumin, which is extremely abundant in the cultures (similar to the situation in serum, which has been well described in the literature [4–6].

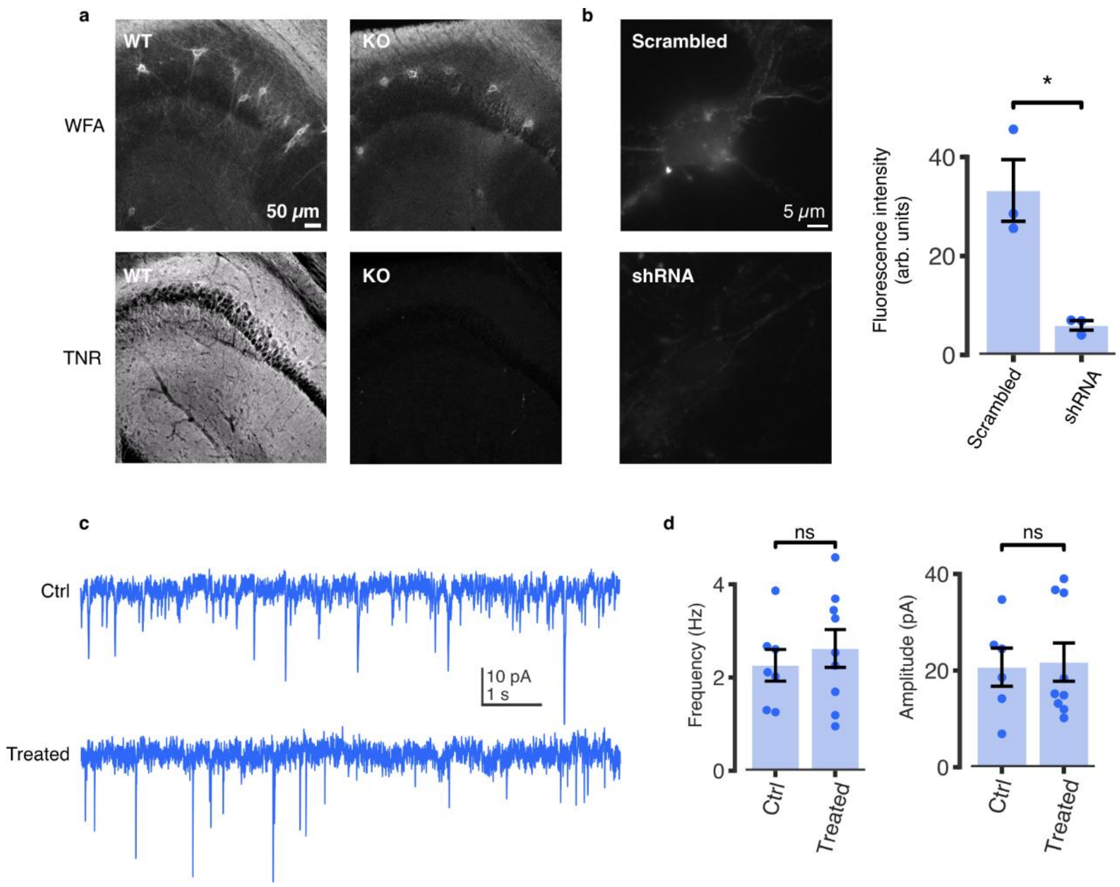

Supplementary Figure 5 Validation of TNR antibodies. a-b, The TNR antibodies used throughout this study were validated in both knock-out and knockdown neurons. **a**, Hippocampal slices of Brevican/TNR KO mice, which were generated by cross-breading of TNR and brevican KO mice [13,14] imaged in epifluorescence microscopy. Top panels: *Wisteria floribunda* agglutin (WFA) staining. Bottom panels: TNR staining. The TNR signal is entirely lost in the KO (right) as compared to wildtype (WT) slices (left). Hippocampal CA2 regions are shown. Scale bar = 50 µm. N = 9 sections from 3 different mice **b**, Neuronal cultures were infected at DIV7 with AAV vectors co-expressing eGFP together with shRNA against TNR, or a scrambled control. Sample images of neurons fixed at DIV21 from scrambled- (top panel) or shRNA-treated (KD) cultures (bottom panel). Scale bar = 5 µm. An analysis of the mean fluorescence intensity (from epifluorescence microscopy experiments) indicates that TNR is significantly reduced in KD cultures, to ~ 20% of the amount in untreated cultures. N = 3 independent experiments, at least 10 neurons imaged per datapoint. Statistical significance was evaluated using a two-sided Student's *t*-test (t = 4.562, **p* = 0.045). The slow turnover of TNR, with a half-life of ~7 days in rat neuronal cultures [3] suggests that ~25% of the TNR should still be present after a knockdown treatment of two weeks. At the same time, this experiment was performed in mouse cultures, for which the AAV vectors were optimized, while other measurements from this work, and from the literature, refer to rat cultures. This implies that turnover values may be somewhat different in this experiment, which was performed solely to verify the antibodies used here. **c-d**, Treatment with TNR antibodies does not affect synaptic transmission. **c**, We measured miniature EPSCs in control rat hippocampal cultures (top) and in cultures treated with TNR antibodies (bottom). Scale bar = 10 pA (vertical axis) and 1 s (horizontal axis). **d**, We analyzed the mEPSC frequency and amplitude in the cultures, and found no significant differences. N = 7 independent experiments for control and 9 for cultures treated with TNR antibodies. Statistical significance was evaluated using two-sided Mann-Whitney U tests (U = 28, *p* = 0.758; U = 27, *p* > 0.999 for the comparison of frequency and amplitude, respectively). Data represent the mean ± SEM, with dots indicating independent experiments.

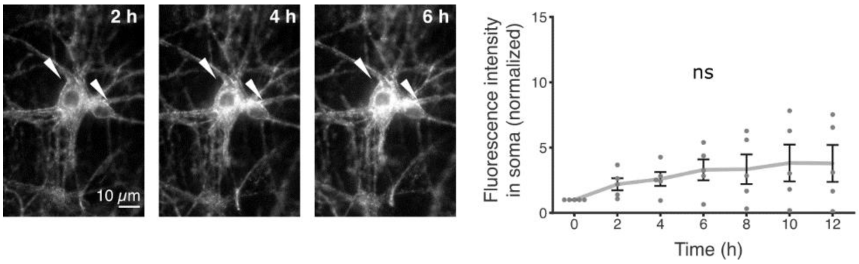

Supplementary Figure 6 TNR molecules endocytose over several hours. All TNR molecules were labeled by applying fluorophore-conjugated TNR antibodies for 1 hour, and were monitored by live epifluorescence imaging over 12 hours. TNR molecules accumulate in the soma over time, but many do not appear to change their location, and neurites remain fully visible over time. Arrowheads indicate cell somas. Scale bar = 10 µm. The plot shows the mean fluorescence intensity in the cell somas, normalized to the t_0 timepoint. A significant increase in the signal over time confirms the suggestion that some proportion of the TNR molecules are endocytosed. N = 5 independent experiments. Statistical significance was evaluated using a two-sided Mann-Whitney U test between the t_0 timepoint and the all sequential timepoints (U = 25, *p = 0.016). Data represent the mean ± SEM.

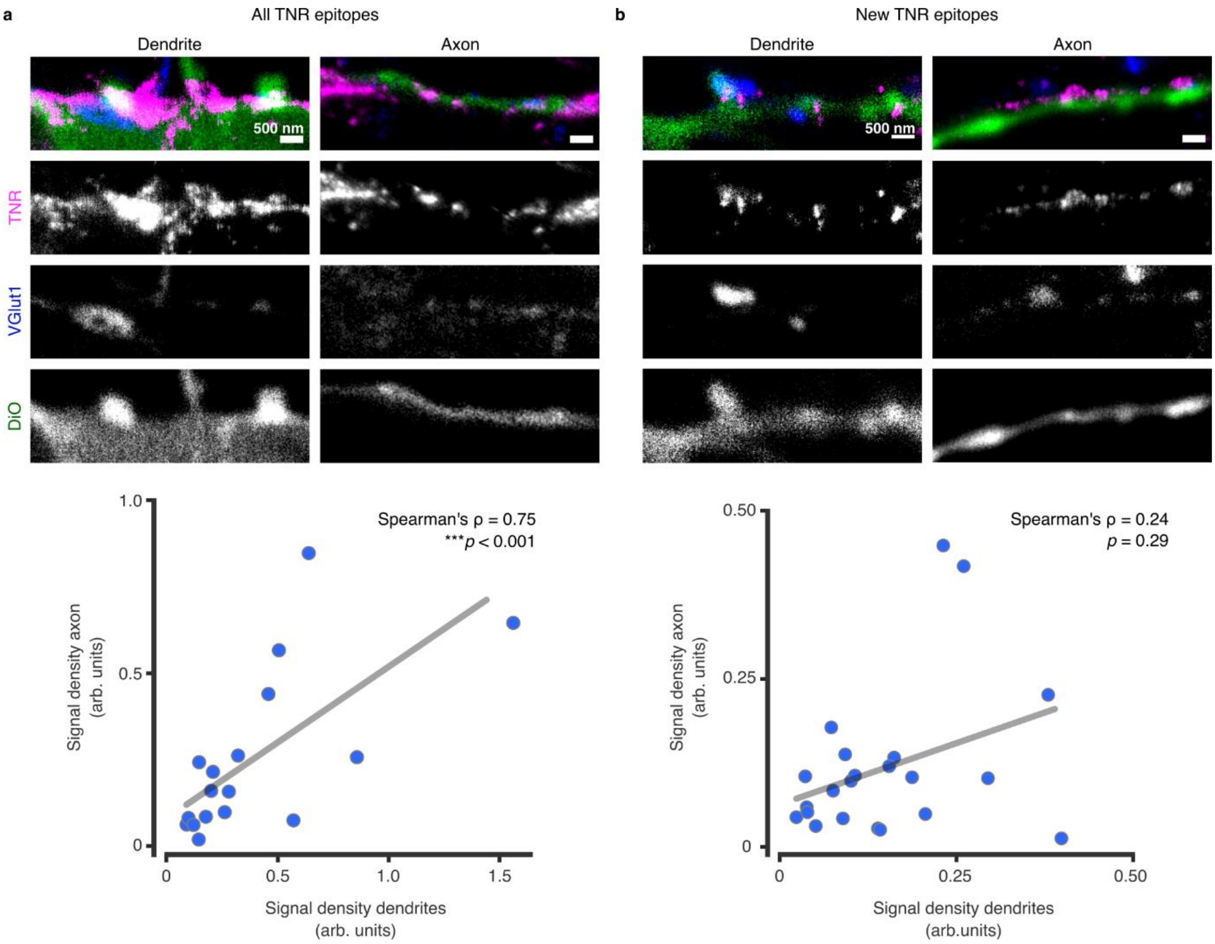

Supplementary Figure 7 The total TNR load of individual neurons is similar for their axons and dendrites, but the load of newly-emerged epitopes is not. We stained individual neurons by incubating the cultures with low levels of DiO crystals (green), which label the plasma membranes of only a few neurons on each coverslip. We then labeled the TNR epitopes (magenta) and the VGlut1-positive boutons (blue) as in Figure 2, and we analyzed the average TNR intensity in the dendrites and the axons of individual neurons. Highly branching neurites with clear spine-shaped protrusions were classified as dendrites, whereas thin, continuous neurites with a substantial number of overlapping VGlut1 puncta were classified as axons. **a**, All TNR molecules in the ECM were labeled. A significant correlation is found between the axonal and dendritic TNR loads. N= 3 independent experiments, with 17 individual neurons imaged; Spearman's ρ = 0.745, ***$p = 0.0006$ (two-sided). **b**, Only the newly-emerged TNR epitopes were labeled. No significant correlation could be determined . N = 3 independent experiments, with 22 individual neurons imaged; Spearman's ρ = 0.237, $p = 0.289$ (two-sided).

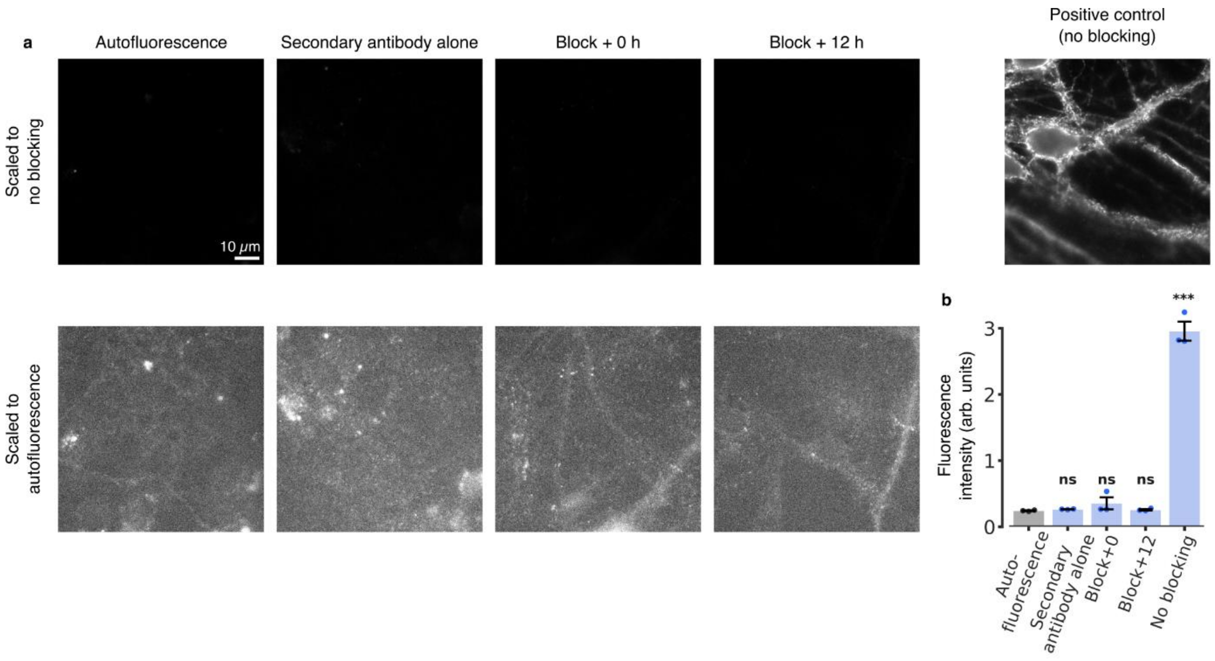

Supplementary Figure 8 The TNR antibodies do not separate from their epitopes in fixed cells at 37 °C. a, We fixed neuronal cultures, and we then blocked their surface TNR epitopes with non-fluorescent antibodies. Immediately afterwards, or after 12 hours, we incubated the neurons with Atto647N-conjugated TNR antibodies. As a control, all TNR epitopes were labeled, by omitting the blocking step. The blocked cultures showed virtually no detectable fluorescence when imaged in epifluorescence microscopy (top panels). In the bottom panels, we enhanced the image contrast, to reveal the outlines of the cells. These are as bright in these images as in negative controls exposed only to Atto647N-conjugated secondary anti-mouse antibodies (leftmost panel). Scale bar = 10 µm. **b**, The analysis of the mean fluorescence intensity confirms that no new TNR epitopes emerge after 12 hours of incubation in fixed neurons, indicating that the blocking antibodies persist on their epitopes, and do not allow the Atto647N-conjugated TNR antibodies to bind. N = 3 independent experiments, at least 10 neurons imaged per datapoint. Statistical significance was evaluated using one-way ANOVA ($F_{4, 10}$ = 120.3, ****p* = 2.06 x 10^{-8}), followed by the Holm-Sidak multiple comparisons test (****p* = 8.20 x 10^{-9}, ****p* = 8.24 x 10^{-9}, ****p* = 1.02 x 10^{-8} and ****p* = 8.11 x 10^{-9}, for the comparison of the ‘autofluorescence’, ‘secondary antibody alone’, ‘block+0’ and ‘block+12’ to the ‘no blocking’ condition, respectively). None of the other conditions were significantly different from the autofluorescence negative control (*p* = 0.993, *p* = 0.716 and *p* = 0.981, for ‘secondary antibody alone’, ‘block+0’ and ‘block+12’, respectively). All data represent the mean ± SEM, with dots indicating individual experiments.

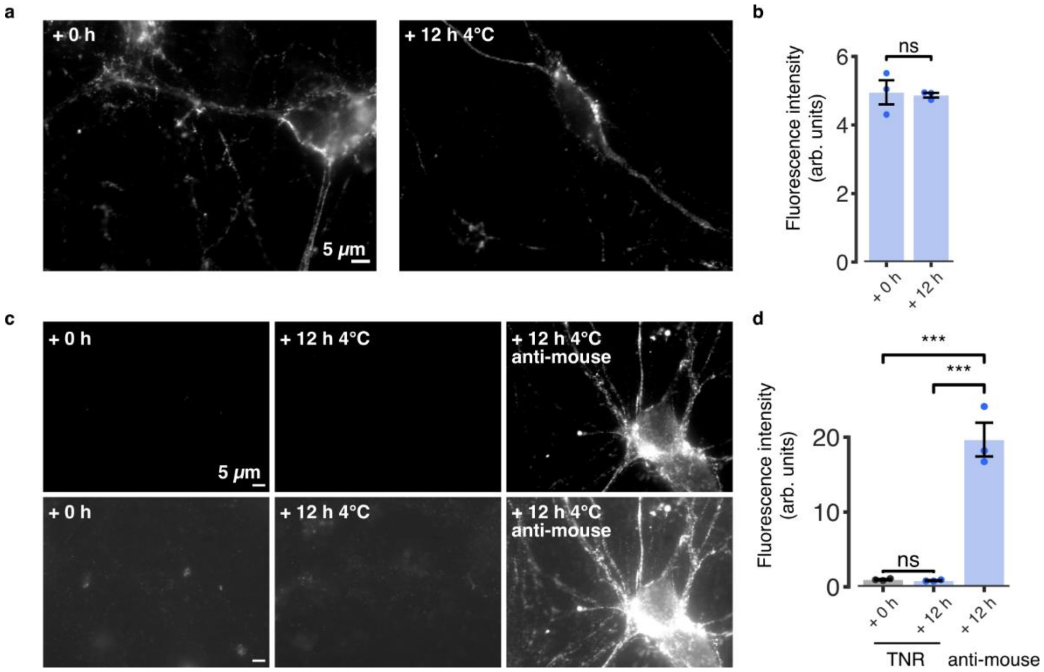

Supplementary Figure 9 The TNR antibodies do not separate from their epitopes in live cells at 4 °C. a, We labeled live neuronal cultures with Atto647N-conjugated TNR antibodies, and imaged them either immediately or following a 12 hour-long incubation at 4°C, in epifluorescence microscopy. Scale bar = 5 µm. **b**, An analysis of the mean fluorescence intensity shows that no significant change in staining is apparent after 12 hours at 4°C, indicating that the antibodies persist on their epitopes. N = 3 independent experiments, at least 10 neurons imaged per datapoint. Statistical significance was evaluated using a two-sided paired *t*-test (t = 0.286, p = 0.802). **c**, We blocked surface epitopes with unlabeled TNR antibodies, and fixed them either immediately or following a 12 hour-long incubation at 4°C. We then labeled the neurons with Atto647N-conjugated TNR antibodies, to reveal the available TNR epitopes, made available by the putative un-binding of blocking TNR antibodies, or with Atto647N-conjugated mouse secondary antibodies, to reveal the unlabeled blocking TNR antibodies. We then imaged the neurons in epifluorescence microscopy. The lower row of panels shows the same images as the top one, with enhanced imaging gain. Scale bar = 5 µm. **d**, An analysis of the fluorescence intensity shows that the staining with TNR antibodies is similar, and extremely low, both immediately and 12 hours after the blocking step. N = 3 independent experiments, at least 10 neurons imaged per datapoint. Statistical significance was evaluated using one-way ANOVA ($F_{2,6} = 69.32$, $p^{***} = 7.14 \times 10^{-5}$) followed by the Holm-Sidak multiple comparisons test (p = 0.945 for the comparison between 'TNR 0 h' and 'TNR 12 h', and p^{***} = 0.0002 for both comparisons to the 'anti-mouse' condition). All data represent the mean ± SEM, with dots indicating individual experiments.

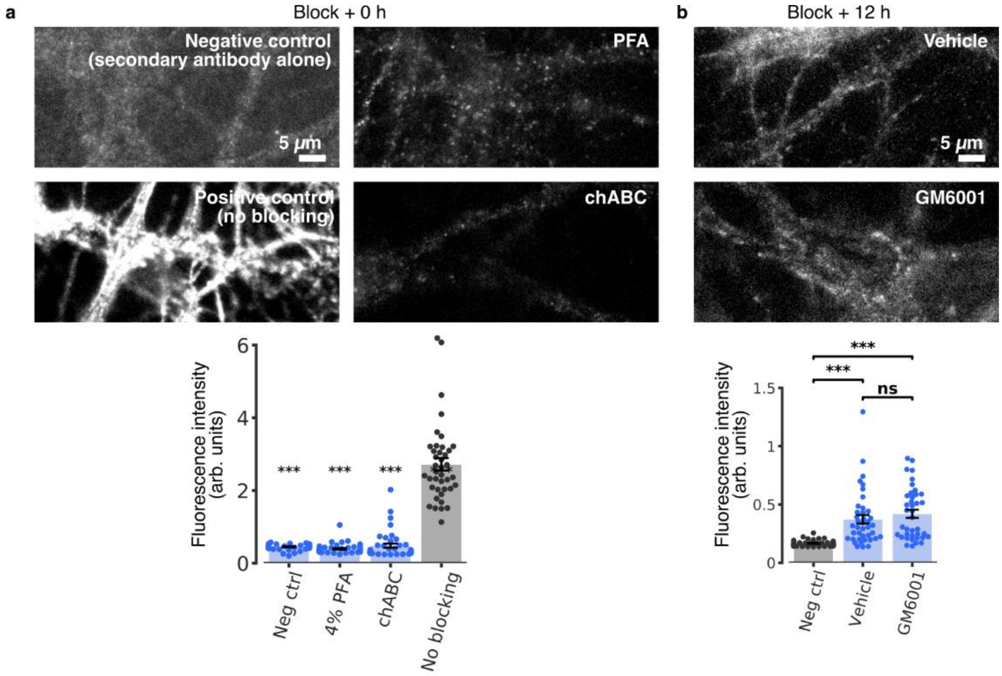

Supplementary Figure 10 The labeled TNRs are not pre-existing extracellular epitopes or the result of cleavage of existing ECM structures. **a**, To test whether the newly-emerged epitopes observed after TNR blocking could represent pre-existing epitopes that simply become available to antibody binding, we blocked surface epitopes with unlabeled antibodies, and then subjected them to several treatments that severely modify the cell surface. The treatments were fixation with 4% PFA or digestion of the surface glycosaminoglycans with chondroitinase ABC. We then added fluorophore-conjugated TNR antibodies to assess the number of epitopes that become available through these procedures. As positive and negative controls we used neurons incubated with TNR antibodies where we omitted the blocking step, or neurons incubated with anti-mouse secondary antibodies, respectively. We then imaged the neurons in epifluorescence microscopy. Scale bar = 5 µm. The graph (bottom panel) indicates that all conditions show substantially less fluorescence than the positive control, and are not distinguishable from the negative control. N = 2 independent experiments for the ‘chABC’ and ‘No blocking’ conditions, and 3 independent experiments for the ‘neg ctrl’ and ‘PFA-fixed’ conditions. 40 neurons were imaged in total per condition. Statistical significance was evaluated using the Kruskal-Wallis test (H_3 = 89.06, ****p* < 0.001), followed by a two-sided Dunn multiple comparisons test for comparing each mean to the ‘no blocking’ condition (****p* < 0.001). **b**, To test whether the newly-emerged TNRs could represent new epitopes that are exposed through the cleavage of existing ECM structures by secreted proteases, we blocked surface epitopes with unlabeled antibodies, and then treated the cultures with GM6001 to block the activity of matrix metalloproteinases (or with DMSO, as a control). We then added fluorophore-conjugated TNR antibodies to assess the amount of epitopes that become available, and imaged the neurons with epifluorescence microscopy. Scale bar = 5 µm. The graph (bottom panel) indicates that drug-treated cultures do not differ significantly from a negative control where neurons were incubated with anti-mouse secondary antibodies. N = 3 independent experiments, 40 neurons imaged in total per condition. Statistical significance was evaluated using the Kruskal-Wallis test (H_2 = 53.34, ****p* < 0.001), followed by a two-sided Dunn multiple comparisons test for comparing the ‘neg ctrl’ condition to ‘vehicle’ or ‘GM6001’ (****p* < 0.001), and ‘vehicle’ to ‘GM6001’ (*p* = 0.95). All data represent the mean ± SEM.

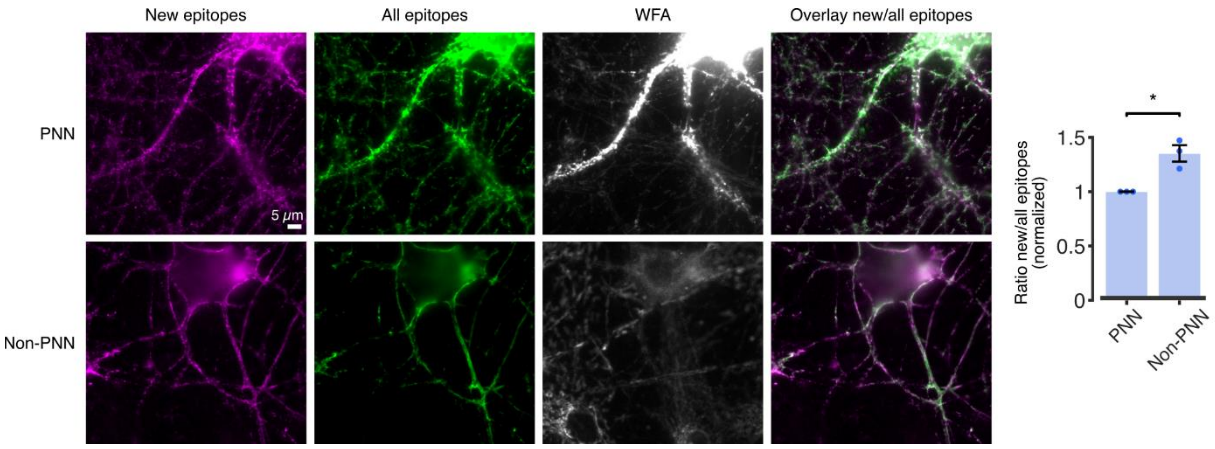

Supplementary Figure 11 TNR uptake and recycling occurs in both PNN and non-PNN-associated neurons. To assess whether the TNR dynamics we observed also hold true for PNN-associated neurons, we checked whether newly-emerged TNR epitopes could be observed in PNN-associated neurons (identified by a co-staining with WFA). We blocked surface epitopes with unlabeled antibodies against TNR, and then labeled newly-emerged TNR epitopes with fluorophore-conjugated TNR antibodies. We then fixed the neurons and incubated them with secondary anti-mouse antibodies, to label all TNR epitopes, and with WFA to label PNNs, before imaging them in epifluorescence microscopy. Newly-emerged TNR epitopes are visible in both PNN-associated (WFA+, top row) and non-PNN-associated (WFA-, bottom row) neurons. Scale bar = 5 µm. An analysis of the fluorescence ratio between new and all TNR epitopes shows that the proportion of newly-emerged epitopes is ~30% smaller for PNN-associated neurons. Nevertheless, this experiment demonstrates that PNN- and non-PNN-associated neurons behave similarly. N = 3 independent experiments, at least 10 neurons imaged per datapoint. Statistical significance was assessed using a two-sided paired *t*-test ($t = 4.663$, $*p = 0.043$). Data represent the mean ± SEM, with dots indicating individual experiments.

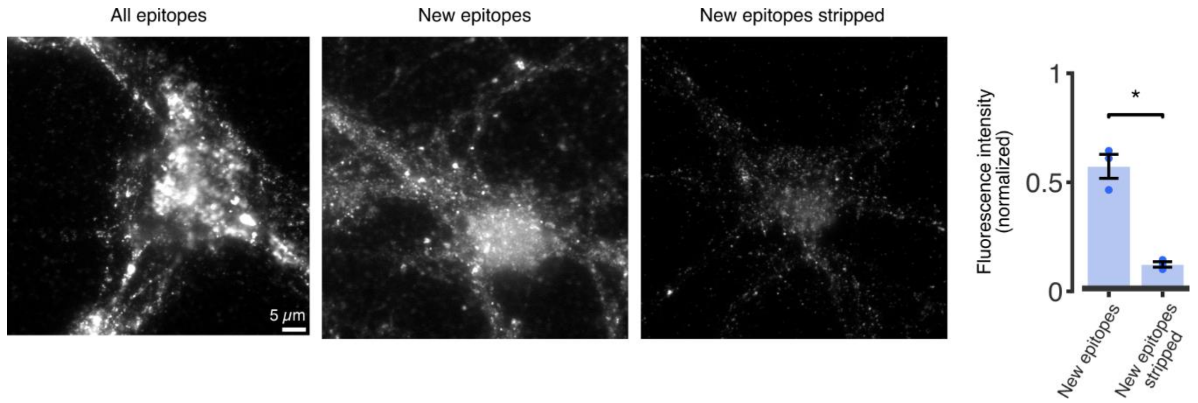

Supplementary Figure 12 TNR dynamics are observed in dissociated hippocampal 'sandwich' cultures. We repeated our 'blocking-labeling' assay in dissociated hippocampal sandwich cultures, in which the neurons are grown at a physical distance above an astrocyte feeder layer. We labeled newly-emerged TNR epitopes 12 hours post-blocking, by applying fluorophore-conjugated antibodies for 1 hour. We then incubated the neurons for an additional 6 hours, to allow for TNR internalization. We stripped the surface-bound epitopes by incubation with proteinase K, and then fixed the neurons and imaged them in epifluorescence microscopy. As controls, we labeled newly-emerged TNR epitopes without stripping the surface-bound molecules, and we also labeled all TNR epitopes by omitting the blocking steps. TNR could be detected in the stripped cultures, suggesting that these molecules are readily endocytosed by the neurons. Scale bar = 5 µm. An analysis of the mean fluorescence intensity (normalized to the 'all epitopes' condition) shows that a fraction of the newly-emerged TNR epitopes is internalized within 6 hours. To confirm that the internalization we observe is not due to unspecific uptake in these cultures, we incubated neurons with Atto647N-conjugated mouse secondary antibodies. This value was subtracted from the mean intensities shown in the plot. N = 3 independent experiments, at least 10 neurons imaged per datapoint. Statistical significance was evaluated using a two-sided paired *t*-test ($t = 8.258$, $^*p = 0.014$). Data represent the mean ± SEM, with dots indicating individual experiments.

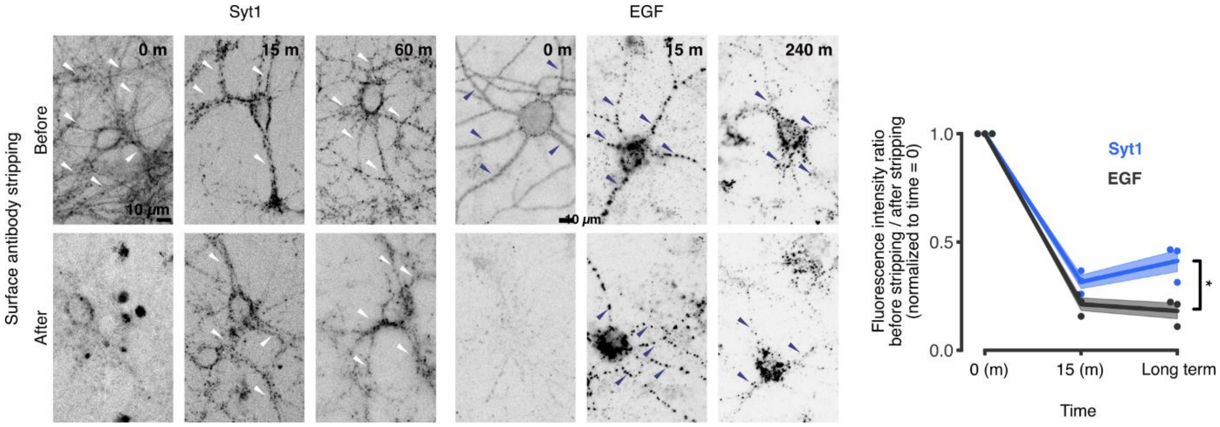

Supplementary Figure 13 Measurement of Syt1 and EGF using the surface antibody stripping assay. As additional controls for the experiment shown in original Fig. 3c, we applied the same surface-stripping assay to two proteins: synaptotagmin 1 (Syt1), which is well known to undergo recycling, and the epidermal growth factor (EGF) receptor, which is endocytosed but does not recycle readily. We incubated the neurons with fluorophore-conjugated antibodies directed against the lumenal domain of Syt1, or with fluorophore-conjugated EGF, and then imaged the neurons in epifluorescence before and after stripping the surface molecules with proteinase K. Immediately after the labeling, the staining was strongly reduced by the stripping, causing neurites to become virtually invisible for both proteins. After 15 minutes, the staining is similar before and after stripping, for both proteins, indicating that they have been endocytosed. Following a longer time period (60 minutes for Syt1; 4 hours for EGF, to account for any long-term recycling of this molecule), a loss of Syt1 from the neurites was observed after stripping, indicating that a portion of Syt1 had resurfaced. This was not the case in EGF-labeled cultures, indicating that they remained inside the cells. We quantified this by reporting the fluorescence ratio between the images taken before and after stripping (normalized to the first timepoint), as in the assay we devised initially for TNR. Scale bar = 10 µm. The graph demonstrates the diverging trends for the two proteins at the latest timepoint. N = 3 independent experiments, 5 images for each condition per datapoint. Statistical significance was evaluated using a two-sided Student's *t*-test (t = 3.782, **p* = 0.019). Data represent the mean (lines) ± SEM (shaded regions); dots indicate the individual experiments.

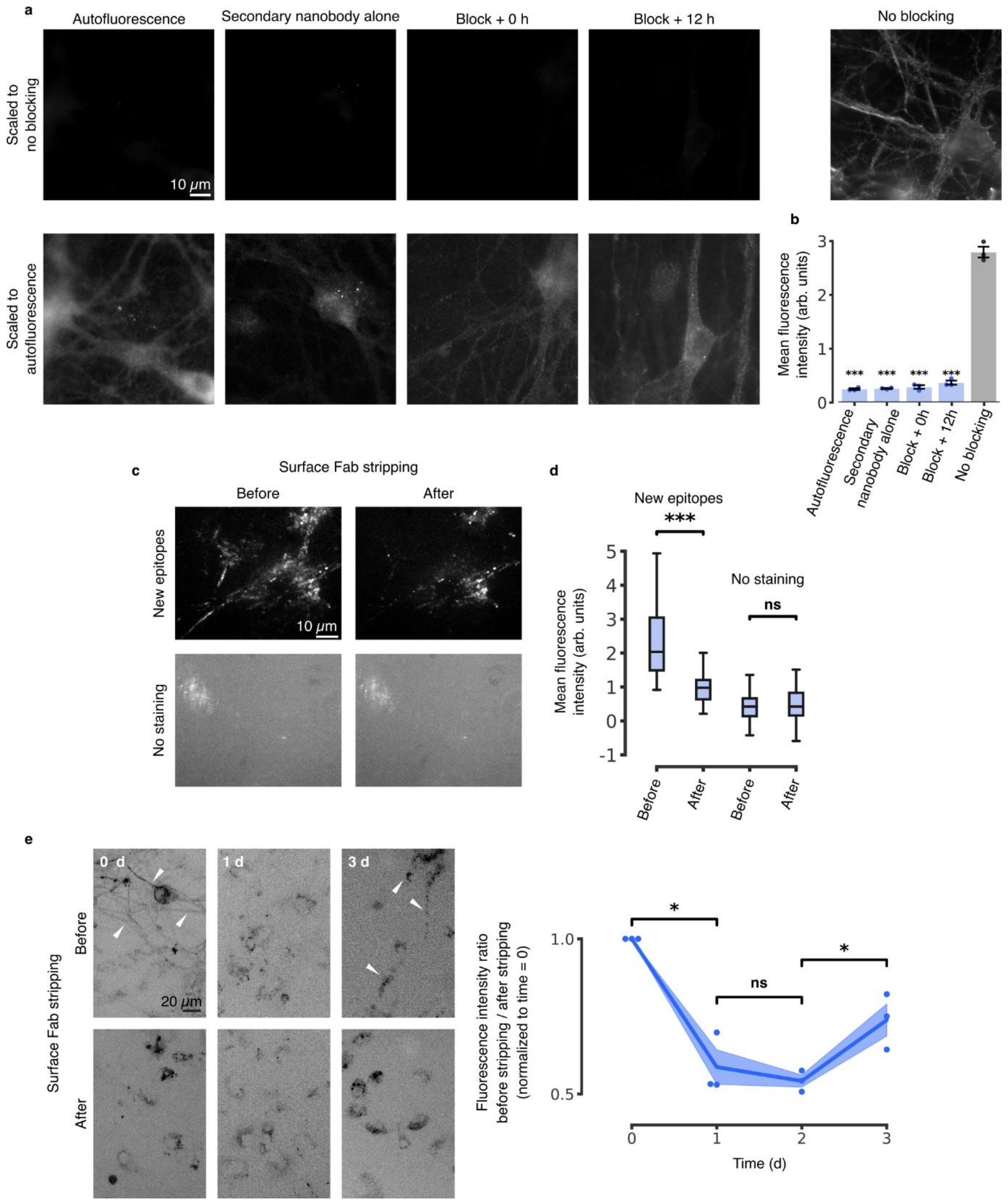

Supplementary Figure 14 The experiments with antibodies are validated with Fab fragments directed against TNR. a, We fixed neuronal cultures, and we then blocked their surface TNR epitopes with Fab fragments directed against TNR, applied together with non-fluorescent anti-mouse nanobodies. Immediately afterwards, or after 12 hours, we incubated the neurons with Fab fragments directed against TNR, applied together with STAR580-conjugated anti-mouse nanobodies. As a control, all TNR epitopes were labeled, by omitting the blocking step. The blocked neurons showed virtually no detectable fluorescence when imaged in epifluorescence microscopy (top panels). In the bottom panels, we enhanced the image contrast, to reveal the outlines of the cells. These are as bright in these images as in negative controls exposed only to STAR580-conjugated secondary anti-mouse nanobodies (leftmost panel). Scale bar = 10 µm. **b**, The application of Fab fragments and STAR580-conjugated anti-mouse nanobodies after 12 hours of incubation in fixed neurons results in no discernible fluorescence signal, indicating that the blocking Fab fragments (coupled to non-fluorescent anti-mouse nanobodies) persist on their epitopes. N = 3 independent experiments, at least 10 neurons imaged per

datapoint. Statistical significance was evaluated using one-way ANOVA ($F_{4,10}$ = 515.909, ***p = 1.56 x 10^{-11}), followed by the Holm-Sidak multiple comparisons test (***p = 5.90 x 10^{-12}, ***p = 6.18 x 10^{-12}, ***p = 5.90 x 10^{-12} and ***p = 9.58 x 10^{-12}, for the comparison of 'autofluorescence', 'secondary nanobody only, 'block+0' and 'block+12' to the 'no blocking' condition, respectively). None of the other conditions was significantly different from the autofluorescence negative control. All data represent the mean ± SEM, with dots indicating independent experiments. **c**, We blocked surface TNR epitopes with Fab fragments directed against TNR, applied together with non-fluorescent anti-mouse nanobodies. Newly-emerged TNR epitopes were labeled 12 hours later with new Fab fragments directed against TNR, applied together with STAR635P-conjugated anti-mouse nanobodies. The Fab fragments bound to surface TNR molecules were then stripped by incubation with proteinase K after a further incubation period of 4 hours. The samples were imaged in epifluorescence microscopy before and after the antibody stripping, and were compared to unstained neurons. Scale bar = 10 µm. **d**, A quantification of the mean fluorescence intensity indicates that a significant amount of surface TNR molecules were stripped at 4 hours after staining, whereas no such reduction was observed for unstained neurons. The amount of intracellular molecules that persisted after stripping was higher than background levels of fluorescence, indicating that a portion of the TNR molecules has endocytosed. n = 28 and 42 regions of interest analyzed from 2 sets of images for the 'new epitopes' and 'no staining' conditions respectively. Statistical significance was evaluated using two-way mixed ANOVA ($F_{1,\,136}$ = 36.58, ***p < 0.001 for the interaction staining x time) followed by the Sidak multiple comparisons test (***p < 0.001 and p = 0.727 for 'before' vs. 'after', for stained and unstained neurons, respectively). Boxes show median (mid-line) and quartiles, and whiskers show minimum and maximum values. Outliers were omitted according to inter-quartile range (IQR) proximity (exceeding 1.5*IQR). **e**, TNR epitopes labeled with Fab fragments recycled back to the plasma membrane. This experiment reproduced the assay presented in Fig. 4c, but using Fab fragments instead of antibodies. We blocked surface TNR epitopes with Fab fragments directed against TNR, applied together with non-fluorescent anti-mouse nanobodies, and labeled the newly-emerged TNR epitopes 4 hours later with new Fab fragments directed against TNR, applied together with fluorophore-conjugated anti-mouse nanobodies. We then measured the fraction present on the surface after different time intervals. To determine this, we imaged the neurons (in epifluorescence) before and after stripping the surface molecules using proteinase K. At day 0 (immediately after labeling), the stripping procedure strongly reduced the staining. The effect was less visible at 1 day after staining, but became again evident at 3 days after staining, indicating that a high proportion of TNR molecules returned to the surface at 3 days after labeling. Scale bar = 20 µm. We quantified this by reporting the fluorescence ratio between the images taken before and after stripping (normalized to the day 0 timepoint). The amount stripped at day 3 is significantly higher than at days 1 and 2. N = 3 independent experiments, 5 images for each condition per datapoint. Statistical significance was evaluated using repeated-measures one-way ANOVA ($F_{1.519,\,3.037}$ = 40.91, **p = 0.007), followed by Fisher's LSD test ('day 0' vs. 'day 1': *p = 0.018; 'day 1' vs. 'day 2': p = 0.522; 'day 2' vs. 'day 3': *p = 0.044). Data show mean (lines) ± SEM (shaded regions), with dots indicating independent experiments. All measurements in this figure refer to neurites, to avoid the bias caused by the higher autofluorescence of the cell bodies, which is evident in such epifluorescence images.

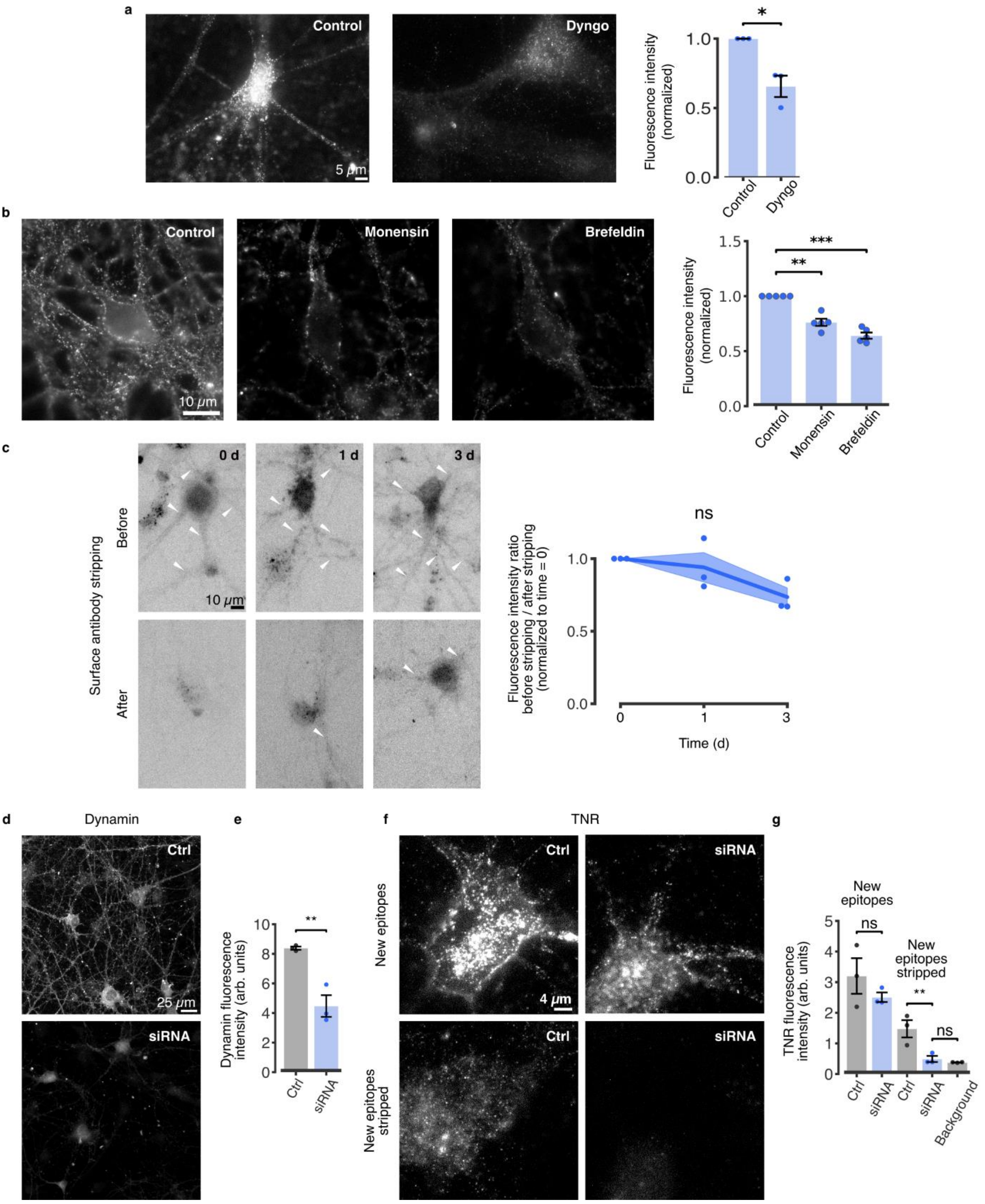
a
Control
Dyngo
5 μm
Fluorescence intensity (normalized)
*
1.0
0.5
0.0
Control
Dyngo
b
Control
Monensin
Brefeldin
10 μm
Fluorescence intensity (normalized)
***
**
1.5
1.0
0.5
0.0
Control
Monensin
Brefeldin
c
0 d
1 d
3 d
Surface antibody stripping
Before
After
10 μm
Fluorescence intensity ratio before stripping / after stripping (normalized to time = 0)
ns
Time (d)
d
Dynamin
Ctrl
siRNA
25 μm
e
Dynamin fluorescence intensity (arb. units)
**
f
TNR
New epitopes
New epitopes stripped
Ctrl
siRNA
4 μm
g
New epitopes
New epitopes stripped
ns
**
TNR fluorescence intensity (arb. units)
Ctrl
siRNA
Background

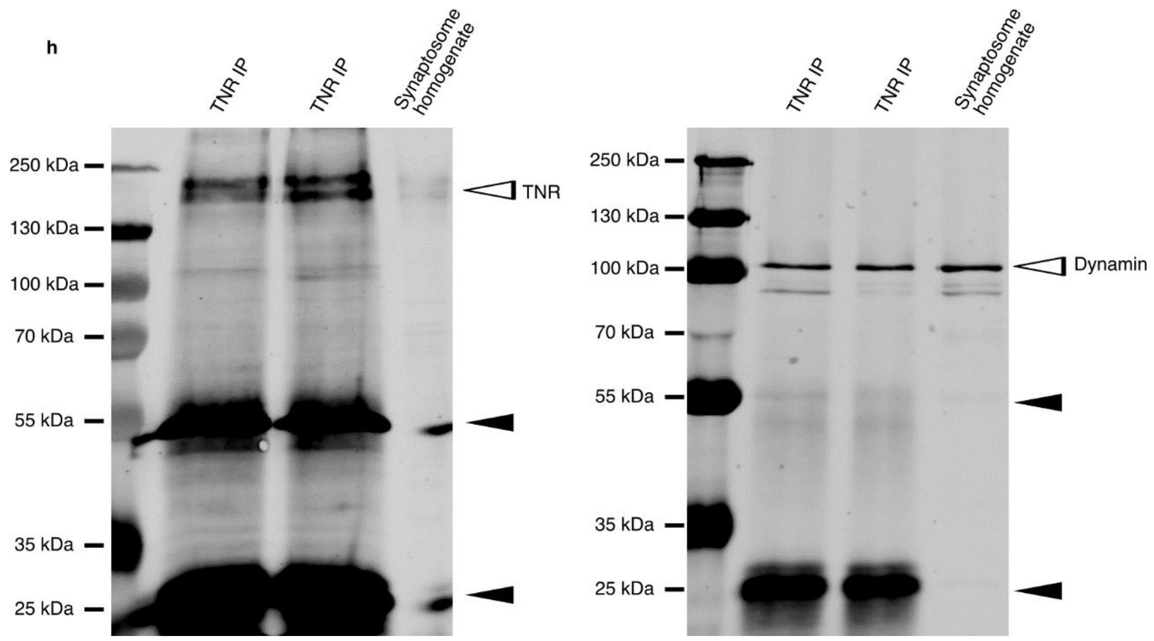

Supplementary Figure 15 Perturbing endocytosis and cellular trafficking reduces TNR recycling. a, Newly-emerged TNR epitopes were labeled 12 hours post-blocking, by the application of fluorophore-conjugated antibodies for 1 hour. Immediately after labeling, dynamin-mediated endocytosis was blocked by incubating the neurons with 30 µM Dyngo® 4a for 2 hours, after which the neurons were stripped by incubation with proteinase K, to reveal only the intracellular epitopes, and imaged with epifluorescence microscopy. The images were compared to control neurons treated with DMSO. Scale bar = 5 µm. The graph shows the mean fluorescence intensity normalized to the control condition in the respective experiment. The drug treatment significantly reduced the amount the internalized TNR epitopes. This treatment cannot be expected to inhibit endocytosis completely in this assay, as the antibodies need to be applied for one hour before the drug, which enables a significant level of endocytosis before the drug can take effect, and because Dyngo at this concentration is not expected to completely remove dynamin function. N = 3 independent experiments, at least 10 neurons imaged per datapoint. Statistical significance was evaluated using a two-sided paired *t*-test (t = 4.076, **p*= 0.015). Data represent the mean ± SEM, with dots indicating individual experiments. **b**, The emergence of new TNR molecules was inhibited by drugs perturbing ER/Golgi traffic. We imaged newly-emerged TNR epitopes (in epifluorescence microscopy) after treatments of 7 hours with DMSO (as a control), with the Golgi-stressing ionophore monensin [7,8], or with the COPI-disturbing inhibitor brefeldin [9]. Scale bar = 10 µm. The graph shows the mean fluorescence intensity normalized to the control condition in the respective experiment. Both drugs reduced substantially the amount of newly-emerged TNR epitopes. N = 4 independent experiments for each condition, at least 10 neurons imaged per datapoint. Statistical significance was evaluated using two-sided paired *t*-tests with Bonferroni correction for multiple comparisons (t = 7.359, ***p* = 0.004 and t = 12.61,****p* < 0.001 for the 'monensin' and 'brefeldin' conditions respectively). Data represent the mean ± SEM, with dots indicating individual experiments. **c**, We repeated the experiment shown in Fig. 4c in the presence of 30 µM Dyngo® 4a, to inhibit dynamin-dependent endocytosis. As expected, stripping significantly decreased the TNR staining immediately after labeling, indicating that the majority of the epitopes are present at the surface. However, this was also apparent at 1 and 3 days after labeling. Scale bar = 10 µm. The graph shows the fluorescence ratio between the images taken before and after stripping (normalized to the first timepoint). A similar ratio across all days suggests that TNR endocytosis is significantly slowed as a result of the drug treatment. N = 3 independent experiments, 5 images for each condition per datapoint. Statistical significance was evaluated using the Kruskal-Wallis test (H_2 = 4.782, *p* = 0.104). Data represent the mean (lines) ± SEM (shaded regions); dots indicate the individual experiments. **d-f**, The endocytosis of recycling TNR epitopes is slowed down by knockdown of dynamin. We performed a triple knockdown of dynamin 1, 2 and 3 using previously described siRNA constructs [10], and assessed the effects on TNR dynamics. **d**, We immunostained control and siRNA-treated neurons with antibodies against dynamin and imaged the cultures with epifluorescence microscopy. The fluorescence signal was visibly reduced in siRNA-treated neurons, demonstrating the effectiveness of the knockdown. Scale bar = 25 µm. **e**, A quantification of the mean fluorescence intensity shows

that the knockdowns reduced the amount of dynamin by ~ 50%. N = 3 independent experiments, 10 images per datapoint. Statistical significance was assessed using a two-sided Student's *t*-test (t = 5.259, $**p = 0.006$). Data represent the mean ± SEM, with dots indicating individual experiments. **f**, We labeled newly-emerged TNR epitopes by applying fluorophore-conjugated antibodies 12 hours post-blocking, and then incubated the neurons a further 6 hours to allow for endocytosis. One group of neurons was fixed immediately after, and the other was subjected to a treatment with proteinase K to remove surface-bound TNR epitopes before stripping. The fluorescence signal was not visibly different when labeling the newly-emerged TNR epitopes in control vs. siRNA-treated cultures (top row). However, the signal when labeling internalized TNR epitopes (following stripping) is significantly reduced in siRNA-treated neurons. Scale bar = 4 µm. **g**, An analysis of the fluorescence intensity confirms that siRNA knockdown of dynamin significantly reduces the internalization of recycling TNR epitopes after 6 hours. The internalized amount in siRNA-treated neurons is not significantly different from the fluorescence background, determined by measuring internalized secondary antibodies in these neurons. N = 3 independent experiments, 10 images per datapoint. Statistical significance was evaluated using one-way ANOVA on log-transformed data ($F_{4, 10} = 38.22$, $***p < 0.001$), followed by Tukey's multiple comparisons test (New epitopes 'ctrl' vs. 'siRNA': $p = 0.86$; New epitopes stripped 'ctrl' vs. 'siRNA': $**p = 0.004$; New epitopes stripped 'siRNA' vs. 'autofluorescence': $p = 0.832$). Data represent the mean ± SEM, with dots indicating individual experiments. **h**, Dynamin co-immunoprecipitates with TNR. We prepared synaptosomes from the cortices of 5 to 6-week-old rats, using a previously established protocol [11]. The synaptosomes were then subjected to a conventional immunoprecipitation procedure, using TNR antibodies. The left panel shows the immunoprecipitation of TNR, while the right panel shows a similar blot, revealing dynamin (using a pan-dynamin antibody). N = 4 independent experiments. The complete lanes are shown in the blots. The IP lanes correspond to 15% of the IP material. For the synaptosome lane, used as a positive control, only 0.05% of the material was run. Filled arrowheads show the bands of the antibodies using for the IP.

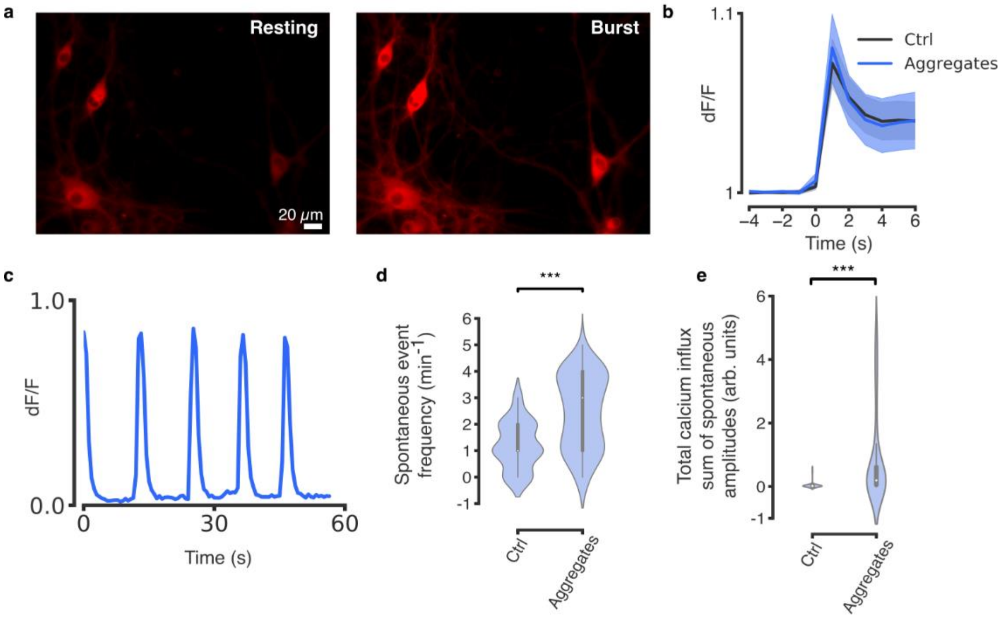

Supplementary Figure 16 Perturbing the recycling pool of TNR molecules affects spontaneous, but not evoked Ca^{2+} activity. To assess the effects the perturbation of the TNR recycling pool on Ca^{2+} dynamics, we incubated dissociated hippocampal cultures with antibody aggregates that bound specifically to newly-emerged TNR. To test whether the TNR pool has an influence on Ca^{2+} dynamics, we measured the Ca^{2+} influx using a neuron-specific fluorescent indicator (red) in live neurons under basal conditions, or following a brief stimulation. **a**, The images show neurons under basal conditions, either at rest (left), or during bursting activity (right). Scale bar = 20 µm. **b**, The Ca^{2+} influx in response to a 1-second, 20-Hz stimulus is not affected by perturbing the TNR pool. N = 4 independent experiments, with 30 and 29 neurons imaged for the 'Ctrl' and 'Aggregates' conditions, respectively. Data show mean (lines) ± SEM (shaded regions). **c-e**, Typical traces from control and aggregate-treated neurons indicate that under basal conditions, perturbing the TNR pool increases the spontaneous firing rate and the total Ca^{2+} influx over time. N = 4 independent experiments, with 74 and 75 neurons imaged for the 'Ctrl' and 'Aggregates' conditions, respectively. Statistical significance was evaluated using two-sided Mann-Whitney U-tests (spontaneous firing rate: U_{145} = 717.5, ****p* = 2.32 x 10^{-14}; total Ca^{2+} influx: U_{56} = 147.5, ****p* = 1.05 x 10^{-5}). Violin plots show the median (white dot), quartiles (box boundaries) and the range of the kernel density estimation (filled area).

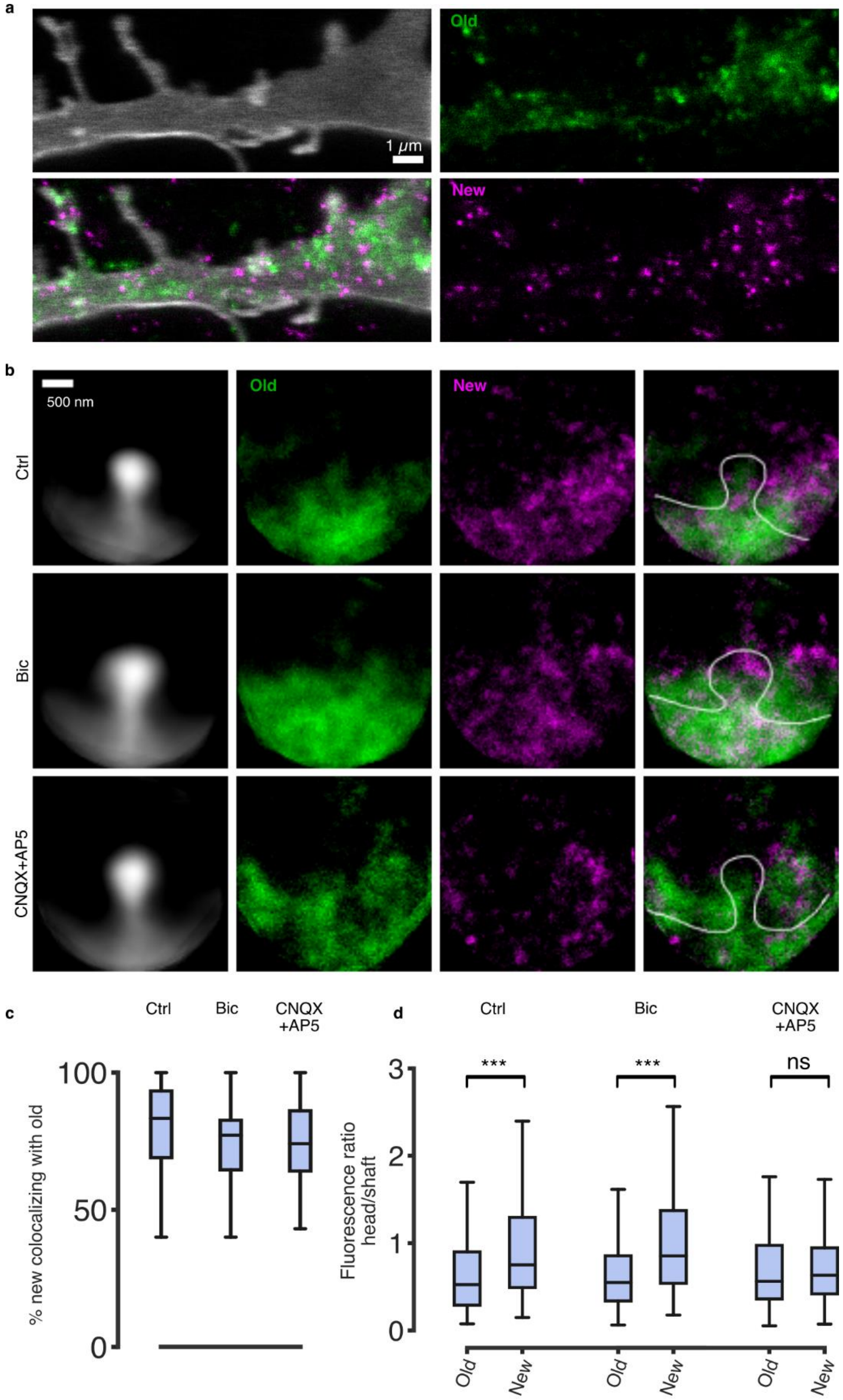

Supplementary Figure 17 New TNR epitopes integrate into the perisynaptic ECM, preferentially near the spine head. To test whether the recycling TNR molecules integrate into the perisynaptic ECM, we performed 2-color-STED super-resolution imaging, to compare the stable and newly-emerged TNR epitopes at the same synapses. In addition, we checked whether the organization of the newly-emerged epitopes is dependent on synaptic activity, by comparing control cultures with cultures treated with bicuculline (40 µM) or with a combination of CNQX (10 µM) and AP5 (50 µM). **a**, An exemplary image of a dendrite. The plasma membrane was visualized by incubating the neurons with DiO. We performed the same 'blocking-labeling' assay as in the

rest of the study, but used fluorophore-conjugated (rather than unlabeled) antibodies against TNR for the blocking step. “Old” and newly-emerged epitopes are typically in the vicinity of each other, albeit they do not correlate strongly, as already suggested in Fig. 2. Scale bar = 1 µm. N = 3 independent experiments. **b**, We imaged many dendrites for each treatment condition and extracted segments containing individual spines. We then aligned these to the same orientation, in order to visualize the average spine under each treatment condition. This visualization showed that the newly-emerged epitopes embed in the ECM, and are especially visible in the vicinity of the spine head. This tendency to localize to the spine head was increased when activity was enhanced with bicuculline, and abolished when activity was reduced with CNQX+AP5. Scale bar = 500 nm. **c**, A quantification of percentage of new TNR epitopes colocalizing with old epitopes showed that the majority of the newly-emerged molecules colocalize with existing molecules, confirming they integrate into the perisynaptic ECM. The remaining epitopes presumably represent endocytosed TNR, found within the dendrites. N = 3 independent experiments, with 88, 114 and 137 synapses analyzed for the ‘Ctrl’, ‘Bic’ and ‘CNQX+AP5’ conditions, respectively. **d**, To test our observation that the new TNR epitopes preferentially localize to the spine head (panel b), we calculated the fluorescence ratio between the spine head and the dendritic shaft at its base, for both the old and the new epitopes. This analysis confirmed that the newly-emerged TNR epitopes preferentially appear in the spine head, whereas the old epitopes are distributed in both the head and the shaft. As we observed in the average images (b), this bias was slightly increased in bicuculline-treated cultures, and was lost in CNQX+AP5-treated cultures. N = 3 independent experiments, with 88, 114 and 137 synapses analyzed for the ‘Ctrl’, ‘Bic’ and ‘CNQX+AP5’ conditions, respectively. Statistical significance was evaluated using two-sided Wilcoxon signed rank tests (‘Ctrl’: W = -1782, ***$p < 0.001$; ‘Bic’: W = -3633, ***$p < 0.001$; ‘CNQX+AP5’: W = -1313, $p = 0.159$). Boxes in panels c-d show median (mid-line) and quartiles, and whiskers show minimum and maximum values. Outliers were omitted according to inter-quartile range (IQR) proximity (exceeding 1.5*IQR).

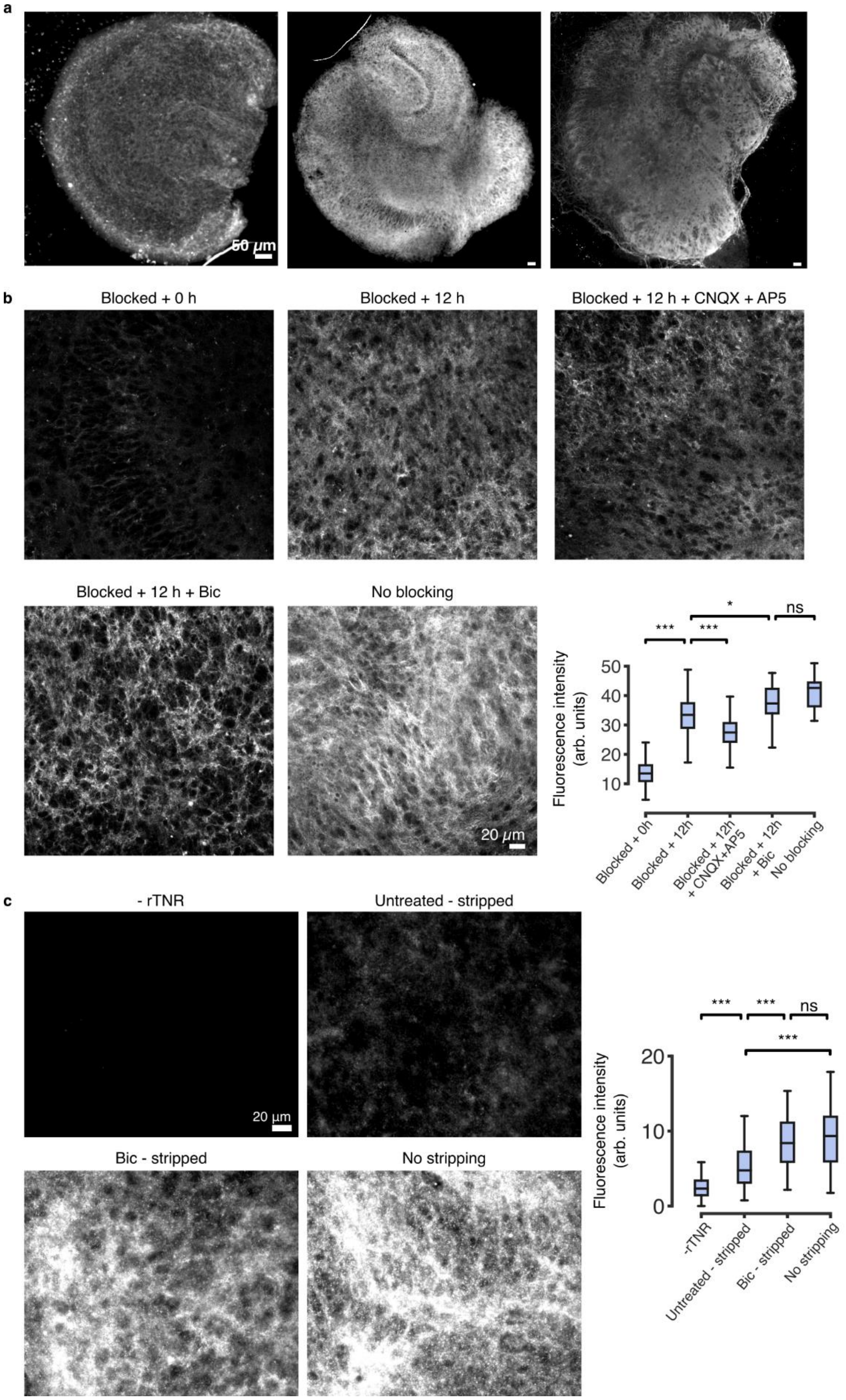

Supplementary Figure 18 TNR dynamics can also be observed in cultured slices. a, Exemplary large field-of-view images of organotypic hippocampal cultured slices, fixed and immunostained for TNR. Scale bar = 50 µm. N = 3 independent experiments. **b**, To test whether Fab fragments can be used to block TNR surface epitopes in a model that is closer to the *in vivo* morphology of neuronal tissue, we applied our ‘blocking-labeling’ assay to organotypic hippocampal slice cultures. We first blocked TNR surface epitopes by incubating live slices with Fab fragments directed against TNR, applied together with non-fluorescent anti-mouse

nanobodies, for 2 hours. Newly-emerged TNR epitopes were labeled with new Fab fragments directed against TNR, applied together with fluorophore-conjugated anti-mouse nanobodies, 12 hours post-blocking. We compared control cultures with cultures treated with bicuculline (40 µM) or with a combination of CNQX (10 µM) and AP5 (50 µM). To test the efficacy of the blocking procedure, we also labeled the newly-emerged TNR epitopes immediately after the blocking step. The blocked slices (‘block + 0 h’) showed little fluorescence when imaged in confocal microscopy, when compared with a full surface labeling (‘no blocking’). Newly-emerged epitopes can be detected 12 hours post-blocking, and their amounts are visibly higher in the presence of bicuculline, and lower in the presence of CNQX + AP5. Scale bar = 20 µm. An analysis of the mean fluorescence intensity confirms that the amount of newly-emerged TNR epitopes is significantly lower following CNQX + AP5 treatment, and significantly higher following bicuculline treatment. N = 3 independent experiments, 10 images for each condition per experiment. Statistical significance was evaluated using one-way ANOVA ($F_{4, 266}$ = 115.8, ****p* = 4.76 x 10^{-57}), followed by Tukey’s multiple comparisons test (‘block+0’ vs. ‘block+12’: ****p* = 9.92 x 10^{-5}; ‘block+12’ vs. ‘block+12+ CNQX+AP5’: ****p* = 0.0002; ‘block+12’ vs. ‘block+12+bic’: **p* = 0.04; ‘block+12+bic’ vs. ‘no blocking’: *p* = 0.492). Boxes show median (mid-line) and quartiles, and whiskers show minimum and maximum values. Outliers were omitted according to inter-quartile range (IQR) proximity (exceeding 1.5*IQR). **c**, We pulsed organotypic hippocampal slices with His-tagged recombinant TNR (rTNR) for 2 hours, and then incubated them a further 6 hours to allow for internalization. Afterwards, the rTNR that remained at the surface of the slices was stripped by incubation with proteinase K. The slices were then fixed and immunostained with anti-His-tag antibodies (to reveal internalized rTNR), and imaged with epifluorescence microscopy. We compared control slices to slices treated with bicuculline (40 µM). To assess the total amount of rTNR and the background fluorescence, we omitted the incubation with proteinase K (no stripping) or the incubation with rTNR (- rTNR), respectively. Scale bar = 20 µm. An analysis of the fluorescence intensity revealed that the amount of rTNR in stripped organotypic slices is significantly higher than the background fluorescence, suggesting that rTNR is internalized in these slices, and that this amount is increased in the presence of bicuculline. In the absence of proteinase K, the amount of rTNR is higher still, showing that a population of the molecules remains at the surface. N = 3 independent experiments, 15 images per condition. Statistical significance was evaluated using the Kruskal-Wallis test (H_3 = 184.1, ****p* = 1.13 x 10^{-39}), followed by a two-sided Dunn multiple comparisons test (’-rTNR' vs. ‘untreated – stripped’: ****p* = 2.47 x 10^{-11}; ‘untreated – stripped’ vs. ‘bic – stripped’: ****p* = 1.28 x 10^{-5}; ‘untreated – stripped’ vs. ‘no stripping’: ****p* = 3.25 x 10^{-7}; ‘bic - stripped’ vs. ‘no stripping’: *p* = 0.519;). Boxes show median (mid-line) and quartiles, and whiskers show minimum and maximum values. Outliers were omitted according to inter-quartile range (IQR) proximity (exceeding 1.5*IQR).

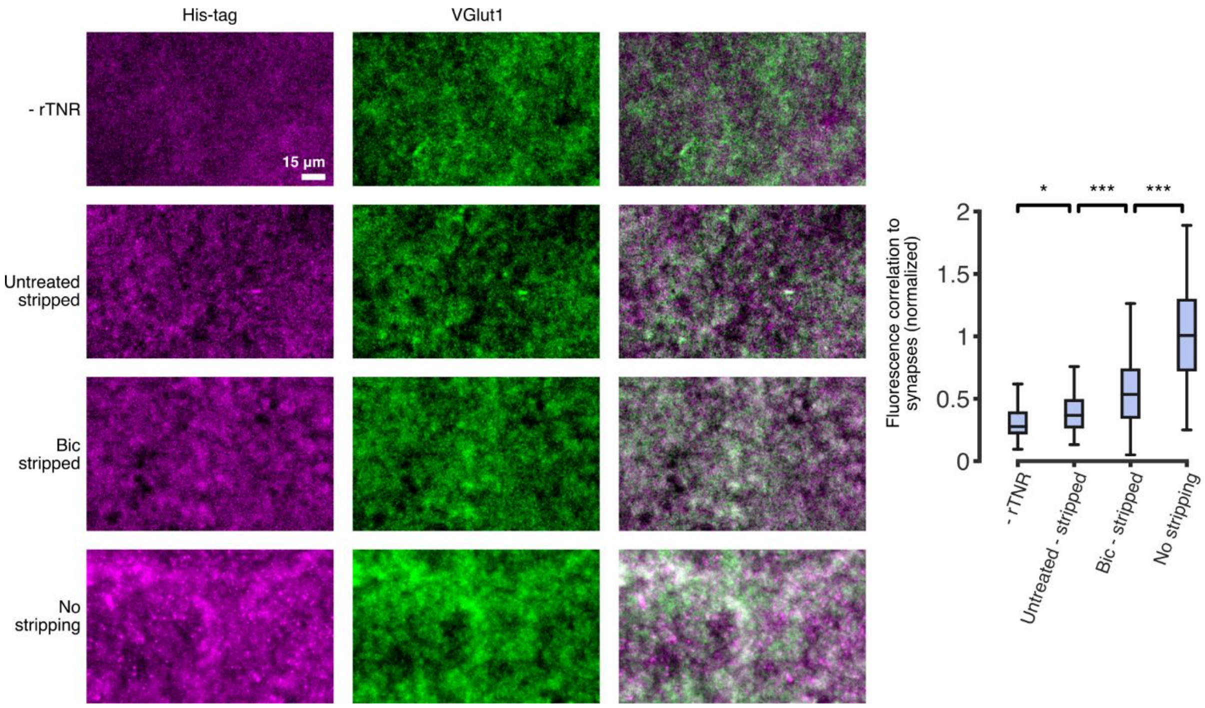

Supplementary Figure 19 His-tagged recombinant TNR is internalized in acute hippocampal slices from rats. To assess whether acute hippocampal slices are capable of internalizing TNR, we incubated slices from 3 week-old rats with His-tagged recombinant TNR (rTNR). Afterwards, the rTNR that remained at the surface of the slices was stripped by incubation with proteinase K. The slices were then fixed and immunostained with anti-His-tag antibodies (to reveal internalized rTNR) and anti-VGlut1 nanobodies (to reveal synapses), and were imaged with epifluorescence microscopy. We compared control slices to slices treated with bicuculline (40 µM). To assess the total amount of rTNR and the background fluorescence, respectively, we omitted the incubation with proteinase K (top row) or the incubation with rTNR (bottom row). Scale bar = 15 µm. We analyzed the pixel-by-pixel correlation between the rTNR and VGlut1 images as a measure for the presence of rTNR in synaptic regions. Unsurprisingly, high amounts of rTNR are found before stripping (as expected from the literature [12]). rTNR was also found in synaptic regions after stripping, and this was enhanced by bicuculline stimulation. N = 3 sections for each condition from 3 different mice, with 20 images acquired per condition. Statistical significance was evaluated using the Kruskal-Wallis test ($H_3 = 179$, ***$p = 1.44 \times 10^{-38}$), followed by a two-sided Dunn multiple comparisons test ('-rTNR' vs. 'untreated – stripped': *$p = 0.035$; 'untreated – stripped' vs. 'bic – stripped': ***$p = 0.0006$; 'bic – stripped' vs. 'no stripping': ***$p = 4.15 \times 10^{-10}$). Boxes show median (mid-line) and quartiles, and whiskers show minimum and maximum values. Outliers were omitted according to inter-quartile range (IQR) proximity (exceeding 1.5*IQR).

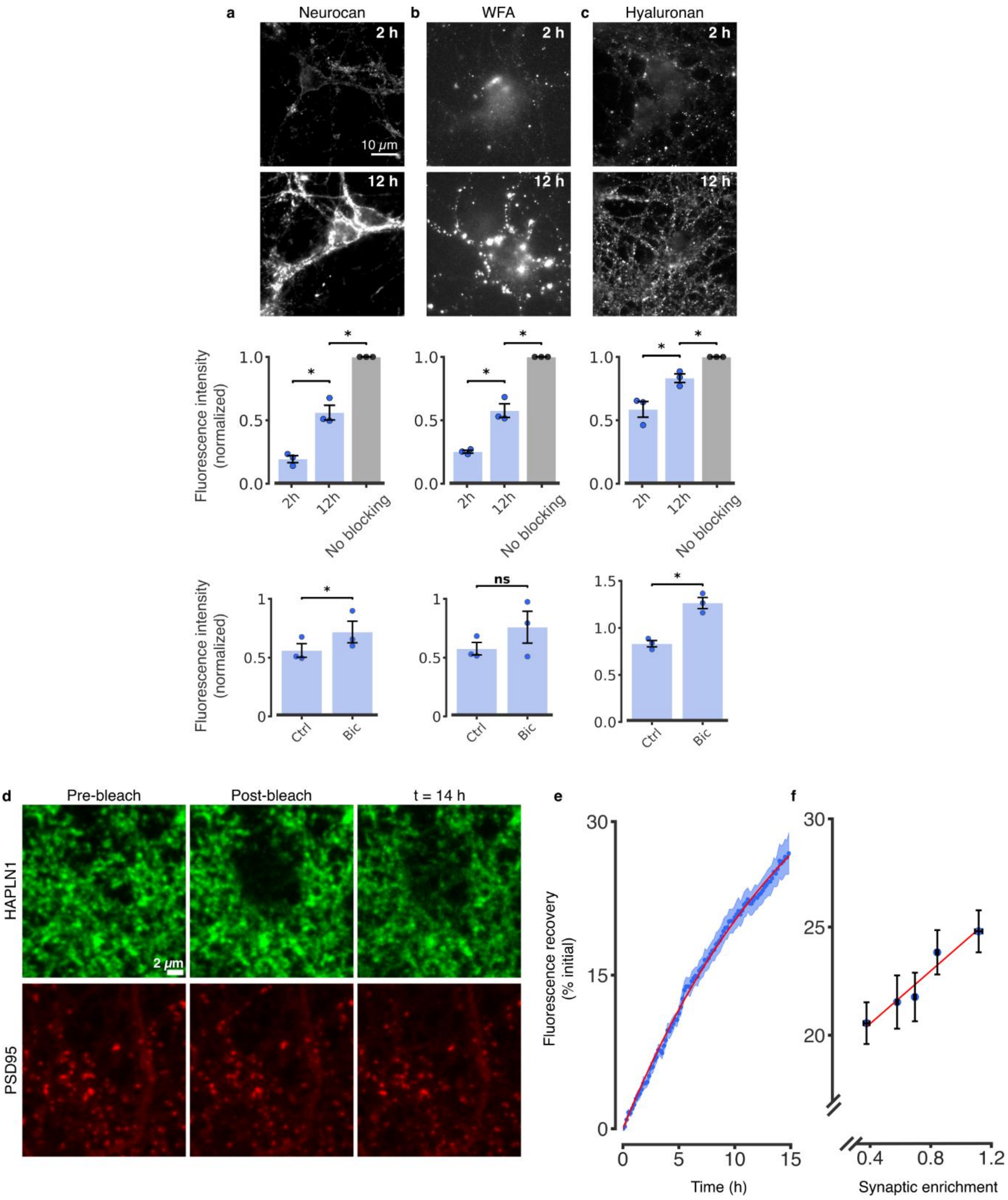

Supplementary Figure 20 Additional ECM molecules are more dynamic than previously presumed, and surface in neurons in an activity-dependent fashion. a-c, Top row: experiments were performed as in Fig. 2b using antibodies for neurocan (panel a) and hyaluronan-binding protein (panel c), or with *Wisteria floribunda* agglutin (WFA), which labels chondroitin-sulfate-bearing proteoglycans (panel b). The cultures were then imaged in epifluorescence microscopy. Scale bar = 10 µm. Middle row: The graphs show the mean fluorescence intensity normalized to a control condition in which the blocking step was omitted. A gradual increase in newly-emerged epitopes was measured, similarly to the dynamics observed for TNR. N = 3 independent experiments for each condition, at least 10 neurons imaged per experiment. Statistical significance was evaluated using repeated-measures one-way ANOVA ($F_{1.005, 2.011}$ = 85.74,**p* = 0.011; $F_{1.013, 2.026}$ = 119.8, ***p* = 0.008; $F_{1.095, 2.190}$ = 42.81, **p* = 0.018) for neurocan, WFA and hyaluronan respectively, followed by Fisher's LSD test (**p* = 0.050 and **p* = 0.017*;* **p* = 0.036 and **p* = 0.016*;* **p* = 0.034 and **p* = 0.038) for the comparisons between '2 h', and '12 h' and '12 h and 'no blocking' conditions for neurocan, WFA and

hyaluronan respectively. Bottom row: we compared control cultures with cultures in which network activity was enhanced by inhibiting $GABA_A$ receptors using bicuculline (40 µM). The graphs show the mean fluorescence intensity normalized to the mean of the bicuculline-treated condition. The bicuculline treatment had a significant effect for 3 molecules. N = 3 independent experiments, at least 10 neurons imaged per datapoint. Statistical significance was evaluated using two-sided paired *t*-tests (neurocan: t = 4.57, *p = 0.045; WFA: t = 1.925, p = 0.194; hyaluronan: t = 4.661, *p = 0.043). Data represent the mean ± SEM, with dots indicating independent experiments. **d-f**, A FRAP-based assay also demonstrates fast ECM dynamics at synapses. **d**, We used FRAP to observe the local turnover dynamics of the hyaluronan-binding protein HAPLN1. The postsynaptic marker PSD95 (red) and HAPLN1 (green) were expressed in neurons, as fluorescent protein chimeras (see Methods). At time 0, regions of ~5 µm radius were bleached in the HAPLN1 channel, using a strong laser pulse. The cells were then imaged once every 10 minutes, for 14 hours, in confocal microscopy. Scale bar = 2 µm. **e-f**, The fluorescence recovery in the bleached regions was analyzed from 14 independent experiments. **e**, A single exponential process was fitted to the fluorescence recovery (red curve), which provided a half-life of approximately 12 hours for the recovery process. The curve shows mean (lines) ± SEM (shaded regions). **f**, To determine whether turnover dynamics differ in synaptic regions, the density of synapses was calculated for the analyzed regions. A strong correlation was found between the percent of fluorescence recovery and synaptic enrichment, indicating that turnover is significantly higher in synaptic regions (R^2 = 0.941, **p = 0.006). Data represent the mean ± SEM.

References

1. Härtig, W., Brauer, K. & Brückner, G. Wisteria floribunda agglutinin-labelled nets surround parvalbumin-containing neurons. *Neuroreport* **3**, 869–872 (1992).
2. Lind, D., Franken, S., Kappler, J., Jankowski, J. & Schilling, K. Characterization of the neuronal marker NeuN as a multiply phosphorylated antigen with discrete subcellular localization. *J. Neurosci. Res.* **79**, 295–302 (2004).
3. Dörrbaum, A. R., Kochen, L., Langer, J. D. & Schuman, E. M. Local and global influences on protein turnover in neurons and glia. *Elife* **7**, (2018).
4. Aroca-Aguilar, J.-D., Fernández-Navarro, A., Ontañón, J., Coca-Prados, M. & Escribano, J. Identification of myocilin as a blood plasma protein and analysis of its role in leukocyte adhesion to endothelial cell monolayers. *PLoS One* **13**, e0209364 (2018).
5. Bottenus, D. *et al.* Preconcentration and detection of the phosphorylated forms of cardiac troponin I in a cascade microchip by cationic isotachophoresis. *Lab Chip* **11**, 3793 (2011).
6. Chart, H., Evans, J., Chalmers, R. M. & Salmon, R. L. Escherichia coli O157 serology: false-positive ELISA results caused by human antibodies binding to bovine serum albumin. *Lett. Appl. Microbiol.* **27**, 76–78 (1998).
7. Dinter, A. & Berger, E. G. Golgi-disturbing agents. *Histochem. Cell Biol.* **109**, 571–590 (1998).
8. Sbodio, J. I., Snyder, S. H. & Paul, B. D. Golgi stress response reprograms cysteine metabolism to confer cytoprotection in Huntington's disease. *Proc. Natl. Acad. Sci. U. S. A.* **115**, 780–785 (2018).
9. Nebenfuhr, A. Brefeldin A: Deciphering an Enigmatic Inhibitor of Secretion. *PLANT Physiol.* **130**, 1102–1108 (2002).
10. Kononenko, N. L. *et al.* Clathrin/AP-2 Mediate Synaptic Vesicle Reformation from Endosome-like Vacuoles but Are Not Essential for Membrane Retrieval at Central Synapses. *Neuron* **82**, 981–988 (2014).
11. Rizzoli, S. O. *et al.* Evidence for early endosome-like fusion of recently endocytosed synaptic vesicles. *Traffic* (2006). doi:10.1111/j.1600-0854.2006.00466.x
12. Morawski, M. *et al.* Tenascin-R promotes assembly of the extracellular matrix of perineuronal nets via clustering of aggrecan. *Philos. Trans. R. Soc. Lond. B. Biol. Sci.* **369**, 20140046 (2014).
13. Brakebusch, C. *et al*. Brevican-deficient mice display impaired hippocampal CA1 long-term potentiation but show no obvious deficits in learning and memory. *Mol. Cell. Biol.* **22**, 7417-7427 (2002).
14. Rauch, U., Zhou, X.H. & Roos, G. Extracellular matrix alterations in brains lacking four of its components. *Biochem. Biophys. Res. Commun.* **328**, 608-617 (2005).
