# Supplementary material for: Extracellular matrix remodeling through endocytosis and resurfacing of Tenascin-R
Source: Nat Commun. 2021 Dec 8;12:7129. doi: 10.1038/s41467-021-27462-7 (PMC8654841; doi:10.1038/s41467-021-27462-7)
Supplement: Supplementary file 3 — Description of Additional Supplementary Files [file 41467_2021_27462_MOESM3_ESM.pdf]

Description of Additional Supplementary Files

File name: Supplementary Movie 1

Description: FRAP-based assay to observe the local turnover dynamics of the hyaluronan-binding protein HAPLN1. Time-lapse video of HAPLN1 (green) and PSD95 (magenta), imaged once every 10 minutes, for 14 hours. Organellelike movement is evident in both channels, indicating ongoing trafficking of proteins. Scale bar = 3 μm.
