# Supplementary material for: Extracellular matrix remodeling through endocytosis and resurfacing of Tenascin-R
Source: Nat Commun. 2021 Dec 8;12:7129. doi: 10.1038/s41467-021-27462-7 (PMC8654841; doi:10.1038/s41467-021-27462-7)
Supplement: Supplementary file 5 — Reporting Summary [file 41467_2021_27462_MOESM5_ESM.pdf]

nature portfolio

Corresponding author(s): Silvio Rizzoli
Last updated by author(s): Oct 19, 2021

Reporting Summary

Nature Portfolio wishes to improve the reproducibility of the work that we publish. This form provides structure for consistency and transparency in reporting. For further information on Nature Portfolio policies, see our Editorial Policies and the Editorial Policy Checklist.

Statistics

For all statistical analyses, confirm that the following items are present in the figure legend, table legend, main text, or Methods section.

n/a	Confirmed	
☐	☒	The exact sample size (*n*) for each experimental group/condition, given as a discrete number and unit of measurement
☐	☒	A statement on whether measurements were taken from distinct samples or whether the same sample was measured repeatedly
☐	☒	The statistical test(s) used AND whether they are one- or two-sided<br>*Only common tests should be described solely by name; describe more complex techniques in the Methods section.*
☐	☒	A description of all covariates tested
☐	☒	A description of any assumptions or corrections, such as tests of normality and adjustment for multiple comparisons
☐	☒	A full description of the statistical parameters including central tendency (e.g. means) or other basic estimates (e.g. regression coefficient) AND variation (e.g. standard deviation) or associated estimates of uncertainty (e.g. confidence intervals)
☐	☒	For null hypothesis testing, the test statistic (e.g. *F*, *t*, *r*) with confidence intervals, effect sizes, degrees of freedom and *P* value noted<br>*Give P values as exact values whenever suitable.*
☒	☐	For Bayesian analysis, information on the choice of priors and Markov chain Monte Carlo settings
☒	☐	For hierarchical and complex designs, identification of the appropriate level for tests and full reporting of outcomes
☐	☒	Estimates of effect sizes (e.g. Cohen's *d*, Pearson's *r*), indicating how they were calculated

Our web collection on statistics for biologists contains articles on many of the points above.

Software and code

Policy information about availability of computer code

Data collection	Data was acquired using Leica Application Suite Advanced Fluorescence 2.7.3.9723, Abberior Instruments Imspector v16.3, NIS Elements 5.02.03 and Olympus CellSens Dimension 2.3, Patchmaster software (HEKA Electronics, Germany).
Data analysis	Custom code written in Matlab2017b and 2019b, Python 3.7.6, icy 1.9.5.1, Graphpad Prism 8 and Excel 2016 was used in this study.

For manuscripts utilizing custom algorithms or software that are central to the research but not yet described in published literature, software must be made available to editors and reviewers. We strongly encourage code deposition in a community repository (e.g. GitHub). See the Nature Portfolio guidelines for submitting code & software for further information.

Data

Policy information about availability of data

All manuscripts must include a data availability statement. This statement should provide the following information, where applicable:
- Accession codes, unique identifiers, or web links for publicly available datasets
- A description of any restrictions on data availability
- For clinical datasets or third party data, please ensure that the statement adheres to our policy

Image data are available from the corresponding author on reasonable request.

Field-specific reporting

Please select the one below that is the best fit for your research. If you are not sure, read the appropriate sections before making your selection.

☒ Life sciences ☐ Behavioural & social sciences ☐ Ecological, evolutionary & environmental sciences

For a reference copy of the document with all sections, see nature.com/documents/nr-reporting-summary-flat.pdf

Life sciences study design

All studies must disclose on these points even when the disclosure is negative.

Sample size	The largest possible numbers of experiments were performed, taking into account the high number of different experimental settings, and is well within the range of typical imaging experiments.<br>We planned to determine major differences between the conditions studied (at least d=1.2 to 1.5), which typically involve "on" or "off" conditions, from drug treatments to biotinylation experiments. For a statistical power of ~80%, our sample sizes should lay around a minimum of 20-25 units. This was achieved in virtually all experiments, as explained in detail in every relevant figure legend. For more details please see:<br>Power failure: why small sample size undermines the reliability of neuroscience.<br>Button KS, Ioannidis JP, Mokrysz C, Nosek BA, Flint J, Robinson ES, Munafò MR. Nat Rev Neurosci. 2013 May;14(5):365-76. doi: 10.1038/nrn3475.
Data exclusions	No experiments were excluded. During data analysis, datapoints were excluded based on standard criteria, as described in the figure legends.
Replication	In general 3 independent experiments were performed, with tens or hundreds of items (synapses, cells, etc.) analyzed. All replications were successful.
Randomization	No comparisons were performed involving samples that could be randomized. All elements in a sample (image) were analyzed, and none were excluded, thereby removing the need to randomly select elements.
Blinding	Data collection for imaging purposes involved selection of fields of view in a color channel that does not show the experimental variable to be measured. This was typically labeled with deep red dyes, which provide the highest imaging precision, but are not seen by the human eye. Therefore, the experimenters chose the fields of view in the green channel, based on other labels (e.g. DiI, GFP, WHA). This implies that the experimenters were inherently blinded to the outcome of each experiment, and no other procedures were necessary.<br>Most analyses relied on automated procedures which are not influenced by the nature of the sample. During selection of regions of interest for analysis within images, the researchers were blind to the experimental condition.

Reporting for specific materials, systems and methods

We require information from authors about some types of materials, experimental systems and methods used in many studies. Here, indicate whether each material, system or method listed is relevant to your study. If you are not sure if a list item applies to your research, read the appropriate section before selecting a response.

Materials & experimental systems

n/a	Involved in the study	
☐	☒	Antibodies
☐	☒	Eukaryotic cell lines
☒	☐	Palaeontology and archaeology
☐	☒	Animals and other organisms
☒	☐	Human research participants
☒	☐	Clinical data
☒	☐	Dual use research of concern

Methods

n/a	Involved in the study	
☒	☐	ChIP-seq
☒	☐	Flow cytometry
☒	☐	MRI-based neuroimaging

Antibodies

Antibodies used	Ankyrin G mouse monoclonal (#75-146; NeuroMab, USA);<br>Calreticulin rabbit polyclonal (#12238S; Cell Signaling, Germany);<br>Caveolin1 rabbit polyclonal (#ab2910; Abcam, United Kingdom);<br>GFAP rabbit polyclonal (#173 002; Synaptic Systems, Göttingen, Germany);<br>Iba1 guinea pig polyclonal (#234 004; Synaptic Systems, Göttingen, Germany);<br>Integrin-Beta1 CD29 hamster antibody (#555003; clone Ha2/5; BD Biosciences, CA, USA);<br>LAMP1 rabbit polyclonal (#ab24170; Abcam, United Kingdom);<br>Myelin basic protein rabbit monoclonal (#78896; Cell Signaling, Germany);<br>NeuN guinea pig polyclonal (#266 004; Synaptic Systems, Göttingen, Germany);

	NeuN mouse monoclonal (#266 011; Synaptic Systems, Göttingen, Germany);<br>Neurocan mouse monoclonal (#N0913; clone 650.24; Merck, Germany);<br>Rab5 rabbit monoclonal (#C8B1; Cell Signaling, Germany);<br>Rab7 rabbit monoclonal (#9367; Cell Signaling, Germany);<br>Rab11a rabbit polyclonal (#2413; Cell Signaling, Germany);<br>Rab11b rabbit polyclonal (#ab3612; Abcam, United Kingdom);<br>Synaptotagmin 1 lumenal domain rabbit polyclonal (#105 103C2; Synaptic Systems, Göttingen, Germany); (manufacturer); validation<br>Synaptotagmin 1 lumenal domain mouse monoclonal (#105 311; Synaptic Systems, Göttingen, Germany); v<br>Tenascin-R mouse monoclonal antibody (#217 011; clone 619; Synaptic Systems, Göttingen, Germany);<br>TGN38 rabbit polyclonal (#T9826, Merck, Germany);<br>VGAT rabbit polyclonal (#131 103; Synaptic Systems, Göttingen, Germany); knock-out validated (manufacturer);
Validation	Ankyrin G mouse monoclonal (#75-146; NeuroMab, USA); validated in Franssen et al., J Neurosci, 2015, https://doi.org/10.1523/JNEUROSCI.2850-14.2015<br>Calreticulin rabbit polyclonal (#12238S; Cell Signaling, Germany); validation in Richter et al., EMBO J, 2018, doi: 10.15252/embj.201695709.<br>Caveolin1 rabbit polyclonal (#ab2910; Abcam, United Kingdom); knock-out validated (manufacturer); validated in Breuer et al., Sci Rep, 2020, doi: 10.1038/s41598-020-73429-x.<br>GFAP rabbit polyclonal (#173 002; Synaptic Systems, Göttingen, Germany); validated in Kaeser-Woo et al., J Neurosci, 2013, doi: 10.1523/JNEUROSCI.5814-12.2013.<br>Iba1 guinea pig polyclonal (#234 004; Synaptic Systems, Göttingen, Germany); knock-out validated (manufacturer); validated in Frühauf et al., Eur J Neurosci, 2020, doi: 10.1111/ejn.14978.<br>Integrin-Beta1 CD29 hamster antibody (#555003; clone Ha2/5; BD Biosciences, CA, USA); validated in Epshtein et al., Mol Metabol, 2017, doi.org/10.1016/j.molmet.2017.07.010<br>LAMP1 rabbit polyclonal (#ab24170; Abcam, United Kingdom); validated in more than 400 publications, for example Rostami et al., J Neuroinflammation, 2020, doi: 10.1186/s12974-020-01776-7.<br>Myelin basic protein rabbit monoclonal (#78896; Cell Signaling, Germany); validated in Zhang et al., PLoS Biology, 2019, doi.org/10.1371/journal.pbio.3000330<br>NeuN guinea pig polyclonal (#266 004; Synaptic Systems, Göttingen, Germany); validated in Ng et al., Nat Biotech, 2020, doi: 10.1038/s41587-020-0742-6<br>NeuN mouse monoclonal (#266 011; Synaptic Systems, Göttingen, Germany); validated in Phillips et al., eLife, 2019, doi: 10.7554/eLife.44182<br>Neurocan mouse monoclonal (#N0913; clone 650.24; Merck, Germany); validation in Shen et al., Glia, 2008, doi: 10.1002/glia.20722.<br>Rab5 rabbit monoclonal (#C8B1; Cell Signaling, Germany); validated in Mutvei et al., Nat Commun, 2020, doi: 10.1038/s41467-020-15156-5<br>Rab7 rabbit monoclonal (#9367; Cell Signaling, Germany); validated in Richter et al., Sci Rep, 2018, doi: 10.1038/s41598-018-33130-6.<br>Rab11a rabbit polyclonal (#2413; Cell Signaling, Germany); validated in Zhang et al., eLife, 2020, doi: 10.7554/eLife.56059<br>Rab11b rabbit polyclonal (#ab3612; Abcam, United Kingdom); validated in Markworth et al., J Neurosci, 2019, doi.org/10.1523/JNEUROSCI.0027-19.2019<br>Synaptotagmin 1 lumenal domain rabbit polyclonal (#105 103C2; Synaptic Systems, Göttingen, Germany); knock-out validated (manufacturer); validation in Courtney et al., Nat Commun, 2019, doi: 10.1038/s41467-019-12015-w.<br>Synaptotagmin 1 lumenal domain mouse monoclonal (#105 311; Synaptic Systems, Göttingen, Germany); validation in Chapman & Jahn, J Biol Chem, 1994, doi.org/10.1016/S0021-9258(17)37523-3<br>Tenascin-R mouse monoclonal antibody (#217 011; clone 619; Synaptic Systems, Göttingen, Germany); validation in this manuscript, in Extended Data Fig. 5<br>TGN38 rabbit polyclonal (#T9826, Merck, Germany); validation in Cattin-Ortolá et al., Traffic, 2017, doi: 10.1111/tra.12507.<br>VGAT rabbit polyclonal (#131 103; Synaptic Systems, Göttingen, Germany); knock-out validated (manufacturer); validation in Hartens et al., J Neurosci, 2008, doi: 10.1523/JNEUROSCI.3887-08.2008

Eukaryotic cell lines

Policy information about cell lines

Cell line source(s)	HEK 293T (Thermo Scientific, #HCL4517)
Authentication	None of the cells used were authenticated.
Mycoplasma contamination	All cells lines tested are negative for mycoplasma contamination.
Commonly misidentified lines (See ICLAC register)	There are no commonly misidentified lines.

Animals and other organisms

Policy information about studies involving animals; ARRIVE guidelines recommended for reporting animal research

Laboratory animals	Rattus norvegicus: Wistar, both sexes, E18 to 6 weeks;<br>Mus musculus, C57BL6/J mice , both sexes, P0-P3; C57BL6/J 5xFAD mice, male, 6-9 months.<br>Housing conditions: 12/12 light/dark cycle (light on from 6 P.M. to 6 A.M), constant temperature (22+/-2°C), 40-60% humidity. For 5xFAD mice, the light cycle was from 9 P.M. to 9 A.M.
Wild animals	None
Field-collected samples	None
Ethics oversight	All animals were handled according to the specifications of the University of Göttingen or DZNE Magdeburg and of the local authorities, the State of Lower Saxony (Landesamt für Verbraucherschutz, LAVES, Braunschweig, Germany) and State of Saxony-Anhalt (Landeswervaltungsamt, Halle, Germany). All animal experiments were approved by the local authority, the Lower Saxony State Office for Consumer Protection and Food Safety (Niedersächsisches Landesamt für Verbraucherschutz und Lebensmittelsicherheit) or by the Ethical Committee on Animal Health and Care and the local authority of the State of Saxony-Anhalt, Germany: Dezernat 33 - Tierschutzdienst (Postfach 39 49, 26029 Oldenburg, Lower Saxony, Germany).

Note that full information on the approval of the study protocol must also be provided in the manuscript.
